# Stimulus-Based ApoE Alzheimer’s Disease Induction Model Using Microglia-Containing Brain Organoids for Drug Discovery

**DOI:** 10.3390/cells15141266

**Published:** 2026-07-14

**Authors:** Nina Y. Yuan, William D. Richards, Kailyn T. Parham, Sophia G. Clark, Connie S. Lebakken

**Affiliations:** Stem Pharm, Incorporated, 2935 S Fish Hatchery Road PMB #235, Madison, WI 53711, USA; nina.yuan@stempharm.com (N.Y.Y.); william.richards@stempharm.com (W.D.R.);

**Keywords:** brain organoid, organoid, assembloid, microglia, neuroinflammation, lipid dysfunction, Alzheimer’s Disease, APOE, APOE4, scRNAseq

## Abstract

**Highlights:**

**What are the main findings?**

- APOE3/3 organoids (NPCs, ECs/MSCs) were generated containing either APOE3/3 microglia or APOE4/4 microglia and supplemented with their respective recombinant ApoE protein (rApoE) with or without amyloid beta 1-42 (Aβ), revealing that rApoE4 induces a neuroinflammatory response regardless of APOE3/3 or the APOE4/4 gene.
- APOE3/3 neuroimmune organoids exposed to a combination of rApoE4 and Aβ (rApoE4 AD model) induce microglial lipid accumulation, pro-inflammatory cytokine and chemokine secretion, neurodegeneration, and an increase in the Aβ42/40 and p-tau181/t-tau ratio.

**What are the implications of the main findings?**

- Alzheimer’s Disease (AD)-like phenotype induction using recombinant ApoE4 protein (rApoE4) with Aβ offers a convenient stimulus-based disease model with a defined timeline and trajectory that is suitable for drug discovery and development.
- Proof-of-concept drug screening found that canonical p38 MAPK inhibitor SB203580 reduced IL-8 secretion, microglial lipid accumulation, and NfL release in the rApoE4 AD neuroimmune organoid model.

**Abstract:**

Alzheimer’s Disease (AD) is a multifaceted progressive neurodegenerative disease characterized by memory deficits and cognitive impairment. The disease is clinically diagnosed by the presence of β-amyloid (Aβ), hyperphosphorylated tau, and neurodegeneration. Animal models serve as an indispensable tool to understanding AD pathogenesis and evaluating potential therapeutic approaches. However, despite the development of more than 200 rodent models, species-specific differences limit the translational relevance. Brain organoids generated from induced pluripotent stem cells (iPSCs) have the potential to bridge the gap between transgenic mouse models and clinical trials in human patients. Herein, we describe a robust stimulus-based neuroimmune organoid model that demonstrates neuroinflammation, neurodegeneration, and lipid dysregulation with AD-relevant pathological markers. Using planar organoids containing neurons, astrocytes, microglia, and vascular cells, we performed in-depth characterization of the induced AD-like phenotype using supernatant proteomic analysis, immunofluorescence staining, NfL and GFAP release, scRNAseq, bulk RNAseq, and pathway analysis both acutely (24 h) and chronically (7 days). Furthermore, we show that the induced neuroinflammation, lipid dysregulation, and neurodegeneration can be ameliorated using small molecules. This defined inducible model system presents an opportunity for drug discovery and development using a complex multicellular brain microenvironment derived from human iPSCs.

## 1. Introduction

It has been estimated that 4.8 million Americans have clinically defined pathological Alzheimer’s Disease (AD) as of 2024 [1,2]. As the population of Americans aged 65 and older is projected to grow from 58 million in 2022 to 82 million by 2050, AD, as an age-related disease, is expected to grow to ~14 million patients and poses a significant social and economic burden [1]. Despite development of effective anti-amyloid antibodies, such as lecanemab and donanemab (Leqembi^®^ and Kisunla™, respectively), these forms of treatment are often inaccessible due to factors including cost and insurance coverage, access to diagnostic and infusion facilities, a lack of local clinical resources, or risk of adverse events [3]. Furthermore, though clinically approved amyloid beta (Aβ) immunotherapies effectively clear plaques, they have had disappointing and variable results in slowing the progressive cognitive decline. Thus, the development of safer and more convenient interventions, as well as the development of therapeutics targeting earlier intervention or alternative pathways for use in combination therapies, is now a priority [4]. Genetic studies have moved the focus from three causal genes (APP, PSEN1, PSEN2) associated with early-onset familial AD (EOFAD) to a large collection of genes that contribute to the overall risk of developing AD. However, disease modeling for AD is still largely performed on EOFAD-related mutations using transgenic mice, such as the 5xFAD or J20, that represent only 5% of all AD patients. The overreliance on these models is believed to have contributed to the particularly high failure rate of AD clinical trials (~99.6%), many of which performed well in these preclinical animal models [5].

As attention has shifted to non-amyloid-related genes that contribute to Sporadic Late-Onset AD (SLOAD), studies reveal involvement in immune response, cholesterol, lipid dysfunction, endocytosis, and vascular factors that contribute to the development of AD (reviewed in [6]). These genome-wide association studies (GWAS) have consistently identified key genetic risk variants for SLOAD in genes associated with microglia [7]. These resident CNS myeloid cells play a multifaceted role in AD pathogenesis, promoting chronic neuroinflammation that exacerbates neurodegeneration [8] while having the capability to both limit [9] but also initiate amyloid plaque formation [10], highlighting their central role in the disease. APOE and TREM2 genes have received particular interest as their function affects the phagocytosis of cellular debris and protein aggregates. Analysis of the recent literature points towards an underlying glial lipid dysregulation which leads to profound alterations in metabolism, phagocytosis, and inflammation. In fact, in addition to the two standard pathological markers—amyloid plaques and neurofibrillary tangles—a third pathological hallmark in AD brain tissue was first described in Dr. Alois Alzheimer’s original report on AD in 1907: “adipose inclusions” or “lipoid granules” [11]. The prohibitive effects of this lipid dysregulation prevent the efficient clearance of cellular debris and protein aggregates which, both directly and indirectly, negatively impact neuronal health [12]. The accumulation of lipids, particularly in microglia, has been of particular interest. These lipid-droplet (LD)-accumulating microglia (LDAM) exhibit heightened production of reactive oxygen species and pro-inflammatory cytokines with a concomitant impaired ability to perform phagocytosis [13]. Lipid homeostasis is a tightly regulated process involving many cell types which orchestrate a dynamic exchange of lipids between cells, both in health and in disease [14]. Clinical studies have shown that significant alterations in lipid metabolism occur in early AD pathogenesis [15] and may precede amyloid plaques and neurofibrillary tangle deposition [16]. Recent advances in modern lipidomics indicate perturbations in the brain lipidome occur in aging, are further altered by cognitive impairment, and have been recently proposed as a biomarker for early AD [15,17,18,19,20,21].

APOE4 (APOE: gene, ApoE: protein) was identified as the strongest risk factor for development of SLOAD [22,23], with accumulating evidence suggesting that APOE4 represents the dominant genetic risk factor that accelerates and amplifies the canonical pathological, biomarker, and clinical trajectory of the disease [24,25,26,27,28]. Recent analysis of the proportion of AD burden attributable to either APOE3 and APOE4 has found that almost all AD (71.5–92.7%) is attributable to these alleles, supporting that the APOE pathways play a key role in AD development [29,30]. The multiple functionally polymorphic variants of APOE are a uniquely human attribute as the gene is monomorphic in other primates and animals [31], including mice. The most recognized role of ApoE is to bind to lipids and transport them, primarily from astrocytes to neurons, to support health, function, and repair but also to support vascular integrity, glucose metabolism, and mitochondrial function. Under normal physiological conditions in the CNS, ApoE is predominantly produced by astrocytes, but under pathological conditions it is produced at high levels by microglia and also by neurons [32,33,34,35]. The ApoE4 variant has a reduced lipid transfer capacity, causing lipid dysfunction and accumulation of glial lipids [36,37]. Furthermore, both animal studies and PET imaging in humans have suggested increased brain inflammation, tau accumulation, and neurodegeneration in APOE4 carriers relative to noncarriers [38,39,40]. Lipid droplets have been shown to play a role in combating oxidative stress and work in drosophila models has shown that reducing lipid droplet formation in glia increases neuronal oxidative cell death [41,42]. However, in the case of microglia, many labs have demonstrated that in both human and mouse cells, reduction in lipid droplet formation in LDAM restores impaired phagocytosis and reduces neuronal damage [43,44,45,46].

The search for an appropriate disease model is an issue that has plagued AD for decades. Postmortem and resected brain tissues are an invaluable resource in research but are limited in quantity and impractical for use in drug discovery and development. Microglia are particularly challenging to study and undergo rapid and profound transcriptional changes only hours after isolation from the brain [47]. Mouse models for AD are widely used but, in many cases, fail to recapitulate both cell autonomous and cell non-autonomous molecular mechanisms of a uniquely human disease [48]. Species-specific divergence among microglia and astrocytes is of particular concern [49,50]. The increasingly popular use of induced pluripotent stem cells (iPSCs) has revitalized the neuroscience field, and many labs have adopted systems for the derivation of neuroectodermal cells which has led to readily commercially available human neurons, astrocytes, and microglia. However, these cells in monoculture do not reflect the complexity of multi-directional dialog that occurs among CNS-resident neurons, astrocytes, microglia, pericytes, and endothelial cells [51,52]. Brain organoids offer an ideal model for the discovery and development of novel therapeutics for CNS disorders, incorporating human-centric discovery while performed in the complex multicellular environment found in the brain. However, traditional methods to generate brain organoids lead to variability in size, uneven distribution of cell types, variability in the breadth of cell types, and formation of a necrotic core, all of which contribute to a lack of reproducibility and characterization. Additionally, a lack of standardization results in substantial organoid-to-organoid variability within and across pluripotent stem cell lines due to the genetic background [53]. This issue is further compounded by the challenge of adding microglia which typically do not incorporate efficiently nor distribute evenly into traditional organoids [54]. Due to the indispensable role of microglia in the brain and their involvement in neurodegenerative disease, their integration and function are critical in disease model development. We have addressed these issues by developing a multicellular neuroimmune organoid model [55] with an accessible and functional microglial population uniformly distributed throughout a planar organoid that allows for more convenient visualization and for the effective distribution of nutrients and treatment regimes. Furthermore, unlike similarly multicellular brain organoid models [56] planar organoids do not necessitate cryosectioning prior to immunostaining, making them especially adaptable to high-content screening.

In this study, we have characterized a novel stimulus-based AD model in a planar neuroimmune organoid system. This model demonstrates both microglial lipid accumulation, neuroinflammation, and neurodegeneration by utilizing recombinant ApoE4 protein (rApoE4) and amyloid beta (Aβ) for disease-state induction. Unlike similar organoid models utilizing either extracts from sporadic AD patients [56,57] or genetic mutation-based models [58,59,60] which both require 1-5 months to develop, this stimulus-based model utilizes well-characterized, readily available reagents and provides robust acute and sustained neuroinflammation over weeklong exposure, allowing for flexible treatment regimens and therapeutic strategies. Furthermore, we found that the pro-inflammatory effect of this model was not reliant on APOE3 or APOE4 microglia donor status and thus could be adapted for use in different genetic backgrounds, an inherent problem when comparing iPSC-derived models. We also demonstrate the utility of this model for unique drug discovery campaigns that offer a multicellular environment and high-content data capture from multiple cell types with more human relevant drug discovery opportunities. Herein, we present a brain organoid model containing neurons, astrocytes, microglia, and vascular-like cells for AD drug screening and development, while characterizing a novel stimulus-based method for inducing an AD-like phenotype.

## 2. Materials and Methods

### 2.1. Cell Culture

iPSC-derived human neural progenitor cells (NPCs) from a male donor (FCDI, Madison, WI, USA; APOE3/3) were maintained in complete STEMdiff Neural Progenitor Medium (NPM, STEMCELL Technologies, Vancouver, BC, Canada), prepared by adding 1 mL of the Neural Progenitor Supplement A (STEMCELL Technologies 05836) and 50 μL of the Neural Progenitor Supplement B (STEMCELL Technologies 05837) to 50 mL of Neural Progenitor Basal Medium (STEMCELL Technologies 05834). When thawing, 10 μM Y27632 (Rock inhibitor; Chemdea NC0407157, Med Chem Express, Monmouth Junction, NJ, USA) was included in the medium and removed day 1 post-thawing. NPCs were cultured on Geltrex (Gibco A1413302, Thermo Fisher Scientific, Waltham, MA, USA) diluted 1:100 with DMEM/F12 (Gibco 11330032). Cells were cultured at 37 °C, 5% CO_2_ atmosphere and passaged at 95% confluent with StemPro Accutase (Gibco A1110501) diluted 1:1 with DPBS (Gibco 14190094). NPCs were assessed for expression of NPC markers SOX2, Ki67 and Nestin by immunofluorescence analysis and are used within 5 passages of the original cell banking.

iPSC-derived human iCell endothelial cells (ECs) (FCDI C1114; APOE3/3) were cultured according to the supplier’s recommendations in VascuLife Basal Medium (LifeLine LL-0003, LifeLine Cell Technologies, Frederick, MD, USA) with VascuLife VEGF LifeFactors. Heat-Inactivated FBS (HyClone SH30071.03HI, HyClone, Logan, UT, USA) was added to the complete media at 10% in place of the supplied FBS. The supplied antimicrobial supplement was not added to the complete media. ECs were maintained on a coating of 3 μg/cm^2^ Fibronectin (Sigma FC010, Sigma Aldrich, St. Louis, MO, USA). Cells were cultured at 37 °C, 5% CO_2_ atmosphere and passaged at 80% confluent with TrypLE Express (Gibco 12605028). Cells are used within 5 passages of receipt from the supplier.

iPSC-derived human iCell mesenchymal stem cells (MSCs) (FCDI R1098; APOE3/3) were cultured in complete MSC medium composed of 0.25X Ham’s F-12 (Gibco 11765054), 0.75X IMDM (Gibco 12440046), 50 μg/mL ascorbic acid (Sigma A8960), 0.5X B-27 Supplement Minus Vitamin A (Gibco 12587010), 50 ng/mL bFGF (R&D Systems 233-FB, Minneapolis, MN, USA), 0.05% BSA (Gibco 15260037), 1X GlutaMAX (Gibco 35050061), 450 μM MTG (Sigma M6145), 0.5X N-2 Supplement (Gibco 17502048), 50 ng/mL PDGF-BB (PeproTech 100-14B, Cranbury, NJ, USA). MSCs were cultured on a coating of 5 μg/mL Fibronectin (Sigma FC010) and 10 μg/mL Collagen I (Gibco A1048301). Cells were cultured at 37 °C, 5% CO_2_ atmosphere and passaged at 80% confluent with TrypLE Express (Gibco 12605028). Cells are used within 5 passages of receipt from the supplier.

### 2.2. Organoid Generation and Culturing

Human neuroimmune organoids were generated from NPCs, microglia, ECs, and MSCs. Except for the microglia, cells were cultured per the cell supplier’s instructions prior to plating onto a polyethylene glycol (PEG)-based hydrogel optimized for this application (proprietary formulation, Stem Pharm, similar to that described in [61,62,63]). iPSC-derived microglia (MG) (APOE3/3: FCDI C1110; APOE4/4: FCDI C1227) were added directly to organoids from cryopreservation. Generation of planar neural organoids have previously been described [55,61,62,63]. Briefly, the hydrogel was polymerized in μ-Plate 96-Well 3D plates (Ibidi 89646) and equilibrated overnight in DPBS and then in NMM prior to plating NPCs. NPCs, MSCs, ECs and MG were plated in serum-free medium at the following time points: NPCs day 0 (25 K/well), MSCs and ECs day 3 (18 K and 1.8 K/well respectively), MG day 14 (12.5 K/well). Organoids were maintained in Neural Maintenance Media (NMM): DF3S (DMEM/F12 (Gibco 11330032) supplemented with 64 μg/mL L-ascorbic acid-2-phosphate magnesium (Sigma A8960), 14 ng/mL Sodium Selenium (Sigma S5261), 543 μg/mL Sodium Bicarbonate (Gibco 25080-094)) supplemented with 1X B-27 Supplement (Gibco 17504044), 1X N-2 Supplement (Gibco, 17502048), 1X GlutaMAX (Gibco 35050061), 1X MEM NEAA (Gibco 11140050), 1X Penicillin–Streptomycin (Gibco 15140122). NMM was supplemented with 5 ng/mL Heat-Stable bFGF (Gibco PHG0367) day 0–5 and 100 ng/mL VEGF (R&D Systems 293-VE) day 3–13. Cultures were fed daily with 50% of medium removed and added back with fresh medium. The organoids were cultured until day 21–23 post plating with NMM prior to stimulation or induction of the models. Further characterization and schematics on planar neuroimmune organoid generation have been previously described [55].

### 2.3. Peptides and Proteins

Aβ peptide was purchased from AnaSpec (AggreSure, AS-72216, Fremont, CA, USA) and recombinant human ApoE4 and ApoE3 was acquired from either Peprotech (350-04, 350-02,) or Abcam (ab123766, ab50242, Cambridge, UK). Both manufacturers produce full-length protein from amino acid 19 to 317 in *E. coli* with >90% purity (SDS-PAGE and HPLC) and <1 EU/µg endotoxin level. APOE3 controls from their respective manufacturers were used to account for possible formulation-derived differences (carriers). Both Aβ and ApoE proteins were aliquoted and stored at high stock concentrations at −80 °C to prevent loss of content to adsorption. LPS (50 ng/mL; Sigma L6529) and IFNγ (50 ng/mL; R&D Systems 285-IF) used for qualification and internal control were likewise aliquoted and stored at high stock concentrations at −80 °C per the manufacturer’s instructions. For all experiments shown, 1 µg/mL of recombinant ApoE protein (rApoE) in combination with 5 µM Aβ was added to the cell culture media and incubated for 24 h. To observe long-term alterations (3–7 days), organoids were fed daily with 50% media change with NMM containing 1 µg/mL of rApoE over a period of up to 7 days. Compounds were aliquoted and stored at high stock concentrations per the manufacturer’s instructions. SB203580 (10 µM; Selleck Chem S1076, Houston, TX, USA), Curcumin (20 µM; Selleck Chem S1848), IKK16 (1 µM; Selleck Chem S2882), Celecoxib (10 µM; Selleck Chem S1261), and TAK-242 (1 µM; Selleck Chem S7455) were tested in organoids with vehicle solvent DMSO control (0.1%; Sigma D2650). Organoids were preincubated with the compounds for 1 h prior to stimulation or induction of the models.

### 2.4. Immunofluorescence Analysis

**Figure 1:**
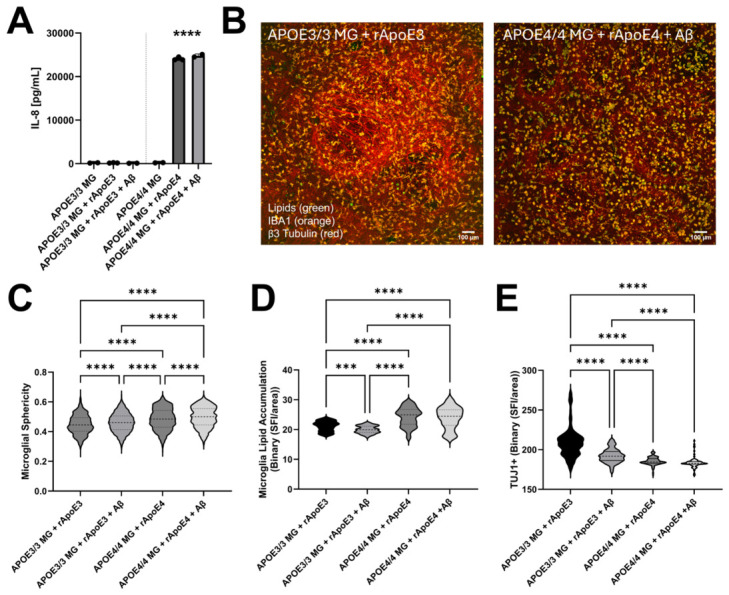
Organoids containing APOE4/4 microglia exposed to rApoE4 exhibit microglial activation, lipid accumulation, and reduction in TUJ1+ neurons. (**A**) Organoids with either APOE3/3 or APOE4/4 microglia exposed to their respective ApoE protein show increase in secreted IL-8 in the cell culture supernatant. (**B**) Immunofluorescence staining of organoid treated with combinations of recombinant ApoE and Aβ. Representative images comparing a control (APOE3/3 MG + rApoE3) versus an “AD”-like phenotype (APOE4/4 MG + rApoE4 + Aβ) at 10X magnification using confocal microscopy. Organoids were stained with IBA1, βIII-Tubulin, and LipidSpot. (**C**–**E**) Quantitative IF analysis confirms significantly increased microglial sphericity (**C**), microglia-specific lipid accumulation (**D**), and decreased fluorescence intensity of βIII-Tubulin+ neuronal staining (**E**). IL-8 measurements (*n* = 2–3 organoids per condition) 24 h after model induction, IF volumetric quantification was performed using 10X confocal imaging, 3 organoids per condition 7 days after model induction. Statistical analysis was performed using one-way ANOVA and Dunnett’s (**A**) or Tukey’s (**C**–**E**) post hoc test; *p*-value levels of significance are as follows: ***, *p* ≤ 0.001; ****, *p* ≤ 0.0001.

Organoids were fixed with 4% paraformaldehyde in DPBS for 1 h and stored in DPBS at 4 °C. Organoid samples were permeabilized and blocked with DPBS containing 10% Donkey Serum (Sigma D9663), 0.2% TritonX-100 (Sigma T9284) and 0.02% Sodium Azide (Fisher S227I,) (Antibody Incubation Solution, AIS) for 1 h. Primary antibodies were incubated overnight at 4 °C and washed three times in DPBS followed by addition of secondary antibodies for incubation overnight at 4 °C and washed three times in DPBS. Alexa Fluor 647 conjugated anti-GFAP (1:200, Abcam ab302828), Anti-IBA1 (1:250, Wako 019-19741), Donkey anti-rabbit AF555 antibody (1:500, Thermo A31572), and Alexa Fluor 647 conjugated anti-β3 tubulin antibody (1:250, RnD Systems IC1195R), and LipidSpot™ 488 (1:1000, Biotium 70065, Fremont, CA 94545, USA) were used for this study. Colocalization measurements of the lipid stain were performed based on positive staining of respective cell-specific markers. Z-stack images were captured on a Nikon AX R confocal microscope (Tokyo, Japan) with a 10× objective. Image processing was performed in Nikon NIS-Elements and Fiji [64]. Binary threshold masking based on positive staining of cell markers was used to extract fluorescence intensity and colocalization measurements using Nikon NIS-Elements. Maximum intensity projection images are presented in representative images (Figure 1B and Supplemental Figure S1). Microglia morphology analysis was performed on images acquired on a Nikon AX R confocal microscope with a 10× objective. Analysis was performed using Fiji (2.9.0) and 3D ImageJ (4.1.7) Suite [65]. Microglia objects were filtered for object volumes of >5000 and <35,000 in calibrated unit. Sphericity in calibrated unit was graphed and analyzed by one-way ANOVA with multiple comparisons. Each treatment analysis used 3 images, with 233–674 microglia present per image.

### 2.5. Neural Organoid Dissociation for scRNAseq

Twelve neuroimmune organoids (day 28) per condition (Table 1) were dissociated using a modified protocol from the Papain Digestion Kit (Worthington Biochemical Corp. LK003150, Lakewood, NJ, USA) and fixation protocols from 10× Genomics (Pleasanton, CA, USA). Organoids were washed with LINC wash buffer (1% Pluronic F-68 (Gibco 24040032), 0.04% BSA (Gibco 15260037) in DPBS) in the culture well. The organoid was transferred to a round-bottomed tube containing the dissociation solution (63% TrypLE Express (Gibco 12605010), 27% Papain (Worthington Biochemical Corp. LK003150), 4.5% DNase (Worthington LK003150), 2 U/μL Protector RNAse (Roche 3335399001, Roche Diagnostics, Indianapolis, IN, USA), 5 μg/mL actinomycin D (Sigma A1410), 10 μM triptolide (Sigma T3652), 27 μg/mL anisomycin (Sigma A9789)). Samples were placed at 37 °C for 40 min with titration every 10 min to dissociate. The samples were centrifuged at 300× *g* for 5 min. The dissociation solution was aspirated, and samples were washed with LINC wash buffer. Samples were passed through a 100 μM cell strainer and centrifuged at 500× *g* for 5 min at 4 °C. The wash buffer was aspirated, and cells resuspended in LINC wash buffer. Samples were counted and checked for viability. Cells were fixed with 2% paraformaldehyde (EMS 15710, Electron Microscopy Sciences, Ambler, PA, USA) for 1 h at room temperature. Equal parts LINC quenching solution (0.1% TRIS; Fisher BP2471500) in LINC wash buffer) was added to the fixed cells. Samples were centrifuged at 500× *g* for 5 min at 4 °C. Solution was aspirated and samples resuspended in wash buffer then counted. Samples were centrifuged at 500× *g* for 5 min at 4 °C. Wash buffer was aspirated, and samples resuspended in LINC freezing buffer (5% DMSO; Sigma D2650), 10% Glycerol (Sigma G5516), 0.2 U/μL SUPERaseIN RNase Inhibitor (Thermo Fisher Scientific AM2694), in LINC wash buffer. Samples were frozen at −80 °C for 24 h prior to moving to liquid nitrogen for long-term storage.

### 2.6. scRNAseq

scRNA-seq was performed at the University of Wisconsin Biotechnology Gene Expression Center. Seurat (v5.3.0) [66] was used for quality control, normalization, sample integration, cell clustering, and visualization. Cells were filtered for features < 12,000, transcripts < 100,000, and mitochondrial percent <10%. Clustering was performed using UMAP. Cell clusters were annotated based on the expression of key marker genes identified for each target cell type. The scRNA-seq data for each cluster was first collapsed using the AggregateExpression() function in Seurat [66], generating bulk-like profiles that represented the pooled expression of all cells in each cluster. The median expression of marker genes associated with each target cell type was then calculated from these bulk-like profiles. Each cluster was subsequently assigned to the cell type with the highest median expression score for its corresponding marker genes. The list of target cell types and their associated marker genes included Excitatory Neurons (*TLX1, TLX3, SLC17A8, CDH9*), Immature Excitatory Neurons (*NEUROG2, NEUROD6*), Inhibitory Neurons (*GAD2, SLC6A5, LAMP5, DLX5*), Radial Glia (*AGT, LRP2*), Dividing Cells (*MKI67, DLGAP5*), OPCs (*OLIG1, OLIG2, SOX10*), Vascular-Related Like (*MGP, TEK*), Astrocytes (*AQP4, CP*), and Microglia (*C1QC, CSF1R*). We used 10x Genomics Loupe Browser v8.1.2 for visualization and differential expression analysis to assist with marker gene identification. GO terms from GO Biological Process (GO: BP) alone were used to generate all results and interpretation. For further information on the methods used for analysis and interpretation, please refer to published reviews [67,68].

### 2.7. Bulk RNA-Seq Sample Collection

Three biological replicates were assessed for each condition, one organoid per biological replicate. Organoids (Day 21) were treated acutely (24 h) with combinations of 1 µg/mL rApoE protein, 5 µM Aβ, and 50 ng/mL of LPS/IFNγ or chronically (7 days) with initial introduction of 1 µg/mL rApoE protein and 5 µM Aβ followed by stimulation with 50 ng/mL of LPS/IFNγ for 24 h prior to harvest. Supernatant was collected and stored at −80 °C and organoids were harvested for RNA using TRIzol Reagent (Invitrogen 15596018). Lysates were processed through a QIAshredder (Qiagen 79656, Hilden, Germany). Samples were frozen at −80 °C prior to RNA purification. RNA was purified using a chloroform extraction protocol utilizing Phasemaker Tubes (Invitrogen A33248), and RNA was purified using the RNA Clean and Concentrator-5 with DNase treatment kit (Zymo Research R1014, Irvine, CA 92606, USA). Samples were eluted in 15 μL of DNase/RNase-free water and stored at −80 °C.

### 2.8. RNA Quantification and Qualification

RNA was quantified following the manufacturer’s protocol for the Quant-iT RiboGreen RNA Assay Kit (Invitrogen R11490). RNA was qualified using a gel electrophoresis protocol as described below. A total of 20–30 ng of RNA was combined with 2× RNA loading dye (NEB B0363S) and denatured at 70 °C for 10 min then placed on ice for 2 min. The ssRNA Ladder (NEB N0362S) was also denatured at 70 °C for 10 min then placed on ice for 2 min. RNA samples and Ladder were loaded on a 1.5% Agarose (Fisher BP160) Gel. The gel was run at 80–100 V for about 60–90 min. The gel was stained using SYBR Gold (Invitrogen S11494) and imaged to determine the integrity of the 28S (~4.8 kb) and 18S (~2 kb) rRNA bands.

### 2.9. RNA Library Prep and Characterization

The Illumina RNA Prep with enrichment, (L) Tagmentation protocol (Document # 1000000124435 v03, Illumina Exome Panel CEX, Oligos 15034575) was followed for the RNA Library Prep. A total of 50 ng of RNA was inputted per sample. The index plate used was the IDT for Illumina—DNA/RNA UD Indexes Set B (Illumina 20025080, San Diego, CA, USA). Throughout the RNA Library Prep protocol, DNA was quantified using the Quant-iT dsDNA Assay Kit, High Sensitivity (Invitrogen Q33120). DNA was qualified using a gel electrophoresis protocol as described below. A total of 20–30 ng of DNA was combined with 6X TriTrack DNA loading dye (Thermo Scientific R1161) and denatured at 90 °C for 2.5 min then placed on ice for 2 min. The GeneRuler 100 bp DNA Ladder (Thermo Scientific SM0243) was not denatured prior to loading onto the gel. DNA samples and Ladder were loaded on a 1.5% Agarose (Fisher BP160) Gel. The gel was run at 80–100 V for 60–90 min. The gel was stained using SYBR Gold (Invitrogen S11494) and imaged to determine the size of the library fragments and to confirm the lack of genomic DNA or primer contaminants.

### 2.10. Bulk RNA-Seq and Analysis

Pooled library samples were sent to the University of Wisconsin Biotechnology Center for bulk RNA-seq. Samples were qualified using a Bioanalyzer (Agilent Technologies, Santa Clara, CA, USA) and analyzed using the TapeStation Analysis Software 5.1 (Agilent Technologies). Pooled samples were sequenced on the NovaSeq X Plus (Illumina). Sequencing data was analyzed through Illumina BaseSpace Sequence Hub (v7.28.0). Samples were concatenated with DRAGEN FASTQ Toolkit (v1.3.1) and aligned using DRAGEN RNA (v4.3.13) with the human reference genome GRCh38. Differential expression analysis was performed using DRAGEN Differential Expression (v4.3.7). Results from DRAGEN Differential Expression were then run through an R pipeline using gprofiler2 (v0.2.2) to perform gene enrichment analysis [69,70]. Genes were used to perform the enrichment analysis for each condition using DESeq2, which removes genes due to low counts or extreme outliers (*p_adj_* ≥ 0.1) prior to downstream analysis. GO terms from GO Biological Process (GO: BP) alone were used to generate all results and interpretation.

### 2.11. Biochemical Assays

Collection of cell supernatant was performed daily in half media changes (50%) or at the experimental endpoint (100%). The collected supernatant was frozen at −80 °C prior to proteomic analysis. Supernatant was analyzed with the Human Cytokine/Chemokine 71-Plex Discovery Assay^®^ Array (HD71) and Human Amyloid Beta and Tau 4-Plex Discovery Assay (HDABT4) (Eve Technologies, Calgary, AB, Canada). Three biological replicates were assessed for each condition, one organoid per biological replicate. Neurofilament light (NfL) and glial fibrillary acidic protein (GFAP) concentrations in the cell culture supernatants were quantified using the Human NEFL SimpleStep ELISA Kit (Abcam ab288171) and the Human GFAP SimpleStep ELISA Kit (Abcam ab288175) per the manufacturer’s protocol, in 384-well format. Samples were diluted 1:100 for the GFAP assay prior to being added to the assay plate. IL-8 concentrations in the cell culture supernatants were assessed with the Lumit IL-8 Immunoassay (Promega CS2032C02, Madison, WI, USA) following manufacturer’s protocol in a 384-well plate format. Measurement of IL-8 induction by 50 ng/mL of LPS/IFNγ stimulation in the organoids was employed as a lot-to-lot quality control metric to demonstrate microglia incorporation and responsiveness to stimulation prior to use in screening. Error bars reflect the standard deviation from the mean.

## 3. Results

### 3.1. APOE3/3 vs. APOE4/4 Microglia

To determine the individual differences between APOE3/3 and APOE4/4 microglia, we generated APOE3/3 organoids (NPCs, ECs, MSCs) and integrated either APOE3/3 or APOE4/4 microglia. In the absence of injury or damage, APOE has been shown to be primarily expressed in astrocytes [71,72]. To account for the absence of the major cellular source of ApoE4, in the otherwise APOE3/3 organoids, we initially used conditioned media from differentiated APOE4/4 NPCs. However, while often used in the literature, this method is not suitable for the reproducibility required in drug screening efforts. Instead, we explored supplementing the organoids with recombinant ApoE (rApoE). Due to the differing lipid-loading affinities between ApoE3 and ApoE4 [73], we used non-lipidated ApoE. Previously published work using recombinant protein has been performed in iPSC-derived neurons and astrocytes in monoculture and in the immortalized mouse BV2 microglia line using concentrations varying from 0.5 to 120 µg/mL [74,75,76], while the quantity secreted naturally from iPSCs in culture has been reported on the lower end of 0.3 µg/mL [75]. Since non-lipidated ApoE is more readily degraded [77,78,79,80], we used an excess (1 µg/mL) to enhance availability of the protein. Additionally, we seeded organoids with amyloid beta (1–42) peptide to determine whether there were discernible differences between APOE3/3 vs APOE4/4 microglia in the presence of Aβ, as a paradigm for early AD pathology.

**Figure 2:**
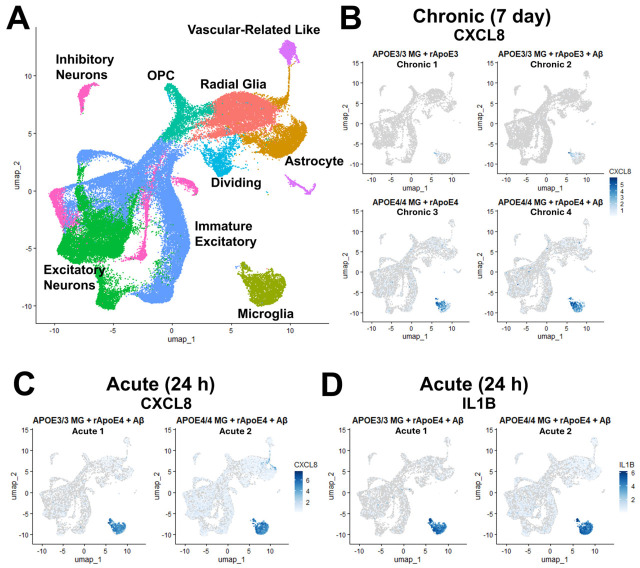
Neuroimmune organoids containing either APOE3/3 or APOE4/4 microglia exhibit a pro-inflammatory response to rApoE4, regardless of genotype. (**A**–**D**) Mapping of scRNAseq data from Stem Pharm’s Neuroinflammatory organoid model (day 28) illustrates distinct CNS-type cell types. (**A**) Annotated UMAP representation of all cells (n = 71,291) from scRNAseq after quality control and batch correction. Organoids from 6 total conditions were sequenced: 4 “chronic” conditions in which organoids were exposed to ApoE protein for 7 days (APOE3/3 MG + rApoE3, APOE3/3 MG + rApoE3 + Aβ, APOE4/4 MG + rApoE4, and APOE4/4 MG + rApoE4 + Aβ; chronic 1-4 respectively) and 2 “acute” conditions in which organoids were exposed to rApoE4 protein for 24 h (APOE3/3 MG + rApoE4, APOE4/4 MG + rApoE4; Acute 1-2 respectively). Cells colored by cell type annotation. Assigned cell types annotated by distinguishing genes as follows: Excitatory (*TLX1, TLX3, SLC17A8, CDH9*), Immature Excitatory (*NEUROG2, NEUROD6*), Inhibitory (*GAD2, SLC6A5, LAMP5, DLX5*), Radial Glia (*AGT, LRP2*), Dividing Cells (*MKI67, DLGAP5*), OPCs (*OLIG1, OLIG2, SOX10*), Vascular-Related Like (*MGP, TEK*), astrocytes (*AQP4, CP*), and microglia (*C1QC, CSF1R*). (**B**–**D**) UMAP visualization of the scRNAseq data set colored by Log2 expression of (**B**,**C**) CXCL8 and (**D**) IL1B. (**B**) Pro-inflammatory cytokine CXCL8 shows higher expression in organoids containing APOE4/4 microglia chronically exposed to rApoE4, (**C**) organoids containing either APOE3/3 or APOE4/4 microglia both show upregulation of CXCL8 when exposed to rApoE4 after 24 h, (**D**) organoids containing either APOE3/3 or APOE4/4 microglia both show upregulation of IL1B when exposed to rApoE4 after 24 h. Clustering and visualization performed using Seurat.

We measured interleukin-8 (IL-8) and microglial sphericity, which have been found increased with both age [81] and in AD patients [82,83,84], to determine microglial activation. Interestingly, we found that organoids containing APOE4/4 microglia secreted high levels of IL-8 and displayed increased sphericity when exposed to rApoE4, while organoids containing APOE3/3 microglia failed to respond to reciprocal levels of rApoE3 (Figure 1A). Acute exposure (24 h) of the rApoE4 protein led to high levels of IL-8 secretion while prolonged exposure (7 days) led to lipid accumulation in the microglia, increased microglial sphericity, and reduction in β3 Tubulin (TUJ1) neuronal staining (Figure 1B–E, additional images in Supplemental Figure S1). To determine the individual cell type contributions to this inflammatory and degenerative phenotype, we performed scRNAseq with six different conditions (Table 1). Organoids (NPCs, ECs, MSCs: APOE3/3) containing either APOE3/3 microglia or APOE4/4 microglia were acutely (24 h) exposed to rApoE4 or chronically (7 days) exposed to their respective rApoE protein with or without amyloid beta. UMAP visualization of scRNA-seq data of all sample sets (*n* = 71,291 cells total) is seen in Figure 2A. An average of 6283 genes were identified in each cell. Cell types were clustered and annotated based on canonical genes described in the figure legend and the distribution of cell types annotated are available in Supplemental Figure S2. In agreement with the increased detection of IL-8 (Figure 1A), we find that the organoids containing APOE4/4 microglia that are chronically exposed to rApoE4 show increased expression of CXCL8 (Figure 2B). Interestingly, we found organoids containing either APOE3/3 or APOE4/4 microglia both similarly exhibited a pro-inflammatory response, increasing expression of CXCL8, IL1B, and IL-6 in response to rApoE4 (Figure 2C,D; Supplemental Figure S3).

**Figure 3:**
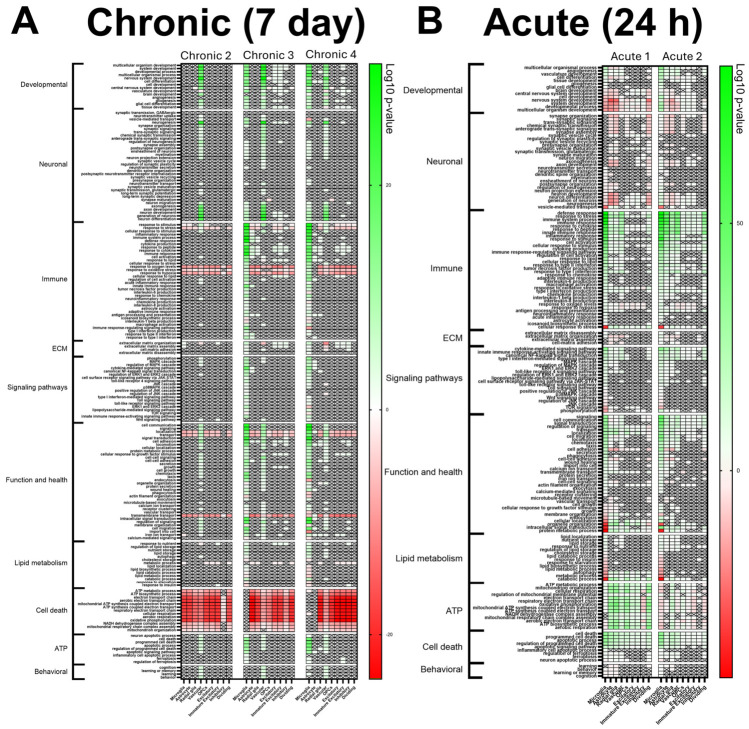
Transcriptomic pathway analysis between organoids containing either APOE3/3 vs. APOE4/4 microglia exposed to their respective ApoE protein shows microglia-specific activation; however, comparison of the response to rApoE4 in either APOE genotype suggests similar pathways underlie the immune and lipid dysfunction in the microglia. Pathway analysis by Gene Ontology enrichment of significantly modulated gene transcriptional changes in annotated scRNAseq data. Organoids (day 28) in sample set Chronic 2 (APOE3/3 microglia + rApoE3 + Aβ), Chronic 3 (APOE4/4 microglia + rApoE4), and Chronic 4 (APOE4/4 microglia + rApoE4 + Aβ) which were exposed to their respective APOE protein over the course of 7 days (**A**) show similarly modulated pathways within the cell populations, with the exception of the APOE4/4 microglia. Organoids (day 28) in sample set Acute 1 (APOE3/3 microglia + rApoE4) and Acute 2 (APOE4/4 microglia + rApoE4) which were acutely exposed to rApoE4 for 24 h (**B**) also show similarly modulated pathways within the cell populations, suggesting that the immune response to rApoE4 share common pathways, regardless of APOE genotype. Representative subset of significantly modulated pathways, (*p* ≤ 0.05) altered by conditions, visualized in a heatmap colored by Log10 *p*-value to a control (Chronic 1: APOE3/3 microglia + rApoE3) for comparison; only statistically significant (≥1.3) Log10 *p*-values are colored and those that did not reach statistical significance are indicated with an X.

**Figure 4:**
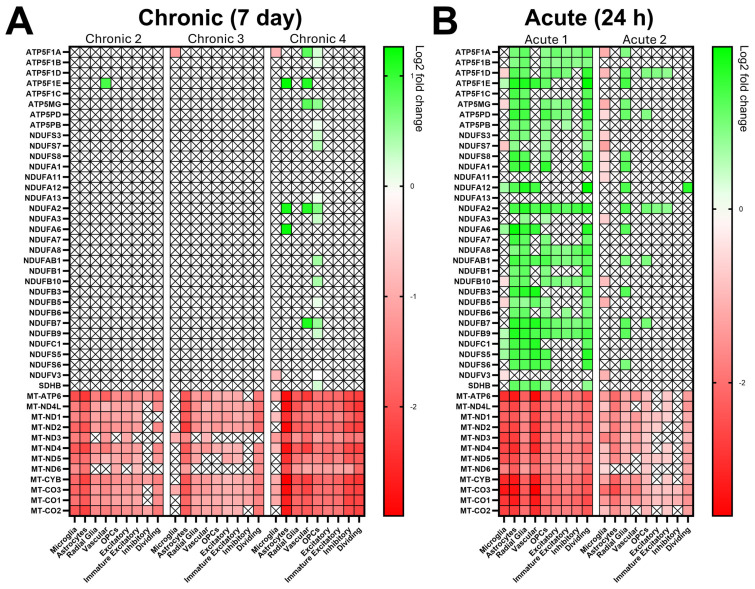
Transcriptomic gene expression of ATP-related genes between organoid builds shows similar trends in mitochondrial dysfunction, despite seeming opposition in the pathway enrichment. Individual differential gene expression from the annotated cell populations in the scRNAseq comparing the (**A**) chronic (7-day) response of organoids containing APOE3/3 vs APOE4/4 microglia exposed to their respective rApoE protein as well as the (**B**) acute response to rApoE4 between organoids containing either APOE3/3 or APOE4/4 microglia. Overall downregulation of mitochondrial ATP-related genes and upregulation of cytoplasmic ATP-related genes are similar between the two sample sets despite pathway enrichment analysis. Differential gene expression of ATP-related genes altered by rApoE4, visualized in a heatmap colored by Log2FC to control (Chronic 1: APOE3/3 microglia + rApoE3) for comparison; only statistically significant (*p* ≤ 0.05) *p*-values are colored and those that did not reach statistical significance are indicated with an X.

Pathway analysis by Gene Ontology (GO) enrichment of the significantly modulated gene transcriptional changes in the annotated cell populations was performed to compare the individual cell responses in the organoid. When organoids containing either APOE3/3 or APOE4/4 microglia were chronically exposed to their respective ApoE protein, there was similar pathway regulation between the samples, particularly in the downregulation of ATP-related pathways caused by either Aβ or rApoE4 (Figure 3A). The primary differences between these organoids were found in the microglia population, showing upregulation of immune response and functional activity-related pathways in these cells, indicating a phenotype primarily driven by microglia. To determine whether this difference was genetic or mediated by the presence of exogenous ApoE4 protein, we compared GO enrichment analysis of organoids containing either APOE3/3 or APOE4/4 microglia that were acutely exposed to rApoE4. This comparison showed remarkably similar results between cell populations, particularly in the microglial cell population, suggesting that the differences were primarily mediated by the exogenous application of rApoE4. However, it is interesting to note that the organoids containing APOE4/4 microglia exhibited more significant changes in the adjacent glial cells with more immune response involvement in the astrocytes, radial glia, and vascular-related populations when exposed to rApoE4, an observation that is consistent with the increased pro-inflammatory phenotype in APOE4/4 cells [85,86,87]. However, the overall response of the organoids containing APOE3/3 microglia versus APOE4/4 microglia to rApoE4 were remarkably similar (Figure 3B). Between the two sample sets, the ATP-related pathways appeared to have the most discrepancies. However, further investigation into the individual gene expression changes in the ATP-related genes in either data set revealed that the overall trends of the significantly modulated genes in the cell populations are the same (Figure 4), with pan-downregulation of mitochondrial related ATP genes (A,B).

This overall gene expression pattern suggests a metabolic shift, which is sustained over time as we observe in the APOE4/4 microglia-containing organoids chronically exposed to rApoE4 and the APOE3/3 microglia-containing organoids seeded with Aβ and chronically exposed to rApoE3 (Figure 3A and Figure 4A). Though Aβ-mediated mitochondrial impairment has been reported previously [88], the addition of rApoE4 as a pro-inflammatory activator appears to compound this effect (Figure 4A) with more significant downregulation of mitochondrial-related genes in 7-day APOE4 MG + rApoE4 + Aβ organoids. Interestingly, when acutely exposed to rApoE4, organoids containing APOE3/3 microglia showed more significant downregulation of mitochondrial genes and upregulation of cytoplasmic genes across all cell populations. Whether this is reflective of the APOE4/4 microglia having some deficiency in metabolic plasticity, as other groups have asserted, requires further exploration [89,90,91].

**Figure 5:**
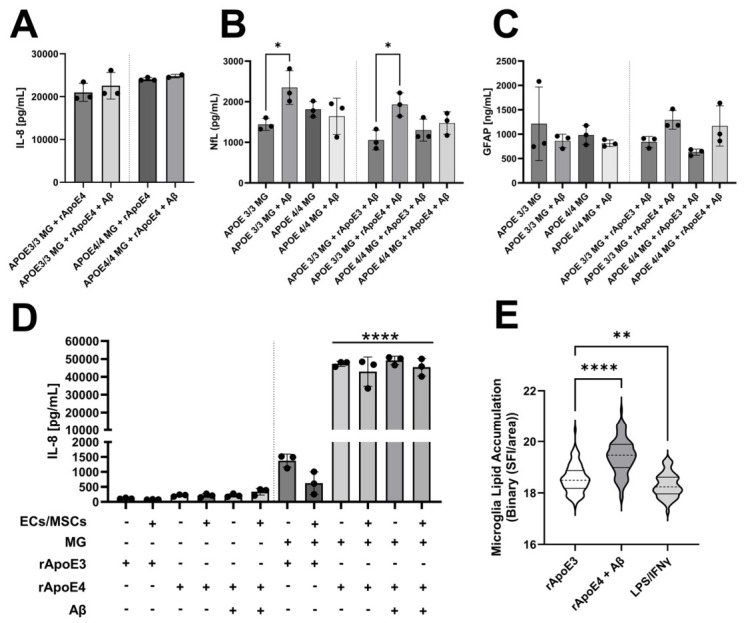
Organoids containing either APOE3/3 or APOE4/4 microglia exposed to rApoE4 secrete pro-inflammatory IL-8; however, only organoids containing APOE3/3 microglia exhibit an increase in NfL release, a clinical biomarker of AD. (**A**) Organoids with either APOE3/3 or APOE4/4 microglia exposed to rApoE4 protein show comparable levels of IL-8 secretion. However, comparison of organoids with either APOE3/3 or APOE4/4 microglia exposed to rApoE4 and Aβ demonstrates that (**B**) only APOE3/3 MG containing organoids release neuronal cell injury marker NfL into the supernatant, despite comparable levels of IL-8 secretion. GFAP release was also measured (**C**), though it did not reach the level of significance via ANOVA; direct *t*-tests between APOE3/3 MG + rApoE3 + Aβ vs APOE3/3 + rApoE4 + Aβ were statistically significant (*p* = 0.0248). (**D**) Characterization of the cell-specific contributions to the IL-8 pro-inflammatory response in the APOE3/3 organoids exposed to rApoE4 + Aβ shows that the disease induction model is primarily microglial-driven. Furthermore, (**E**) microglial lipid accumulation is also induced, as compared to induction with LPS + IFNγ. ELISA measurements includes biological replicates only (*n* = 3 organoids per condition) 24 h after model induction; IF volumetric quantification was performed using 10× confocal imaging using 2 organoids per condition 3 days after model induction. Statistical analysis was performed using one-way ANOVA and Tukey’s (**A**–**C**,**E**) or Dunnett’s post hoc test (**D**); *p*-value levels of significance are as follows: *, *p* ≤ 0.05; **, *p* ≤ 0.01; ****, *p* ≤ 0.0001.

Due to the similarity in the transcriptional response and pathway regulation between either organoids containing APOE3/3 or APOE4/4 microglia, we considered whether rApoE4 + Aβ could be used as an AD-relevant exogenous stimulation, much like how LPS, poly IC, or CoCl_2_ is utilized [92]. These induced (or stimulus-based) models increase reproducibility and circumvent any functional differences that may exist between the response of APOE3/3 vs APOE4/4 microglia due to other variations in the genetic background of the donor cells [93,94]. Comparing further, we analyzed functional readouts of neuroinflammation (IL-8 release) and neurodegeneration (NfL release). The levels of IL-8 secreted among the organoids containing either APOE3/3 or APOE4/4 microglia when exposed to rApoE4 were comparable and were not significantly different (Figure 5A). However, importantly, we found that the release of NfL, a well-regarded biomarker of neurodegeneration [95,96], only occurred in organoids containing APOE3/3 microglia (Figure 5B). It is known that APOE4/4 microglia exhibit deficiencies in phagocytosis and pruning [40], as other studies have shown that crosstalk between APOE3 neurons and APOE4 microglia is impaired [37]. Inversely, whether the introduction of the APOE4/4 microglia led to immediate NfL release (microglia allowed to integrate for 7 days) prior to testing was not assessed, as the relative baseline detection levels of NfL were higher in the organoids containing APOE4/4 microglia. Further study into this genotypic difference would be necessary to interpret the significance of this finding. Nevertheless, in both the absence and presence of recombinant ApoE, we find that organoids containing APOE3/3 microglia release significantly higher levels of axonal injury marker NfL when exposed to Aβ, compared to organoids containing APOE4/4 microglia. As the release of NfL is a hallmark of neurodegeneration and a clinically relevant AD biomarker, this significant release in the APOE3/3 organoids upon disease induction is an important aspect for adapting multiplex assays for drug development in organoid systems which have a variety of cell types. GFAP release, another clinically relevant AD marker for astrocytic reactivity [97,98], was also measured (Figure 5C) with increased release in organoids containing APOE3/3 microglia compared to APOE4/4 microglia (*t*-test 0.0248 versus 0.0907, respectively). To assess cell-specific contributions to the high induction of IL-8, organoids were constructed with or without vascular-related ECs/MSCs (APOE3/3) and with or without MG (APOE3/3) and found that the induction was primarily mediated by the presence of microglia (Figure 5D). Finally, we also found the accumulation of lipids that we observed in APOE4/4 microglia-containing organoids (Figure 5E) also occurs in the APOE3/3 microglia-containing organoids following induction with rApoE4 + Aβ and is not induced by LPS/IFNγ. However, it should be noted that previous studies performed in murine microglial monoculture have shown that LPS treatment led to accumulation of microglial LDs [99]. We did not observe this in our human organoid model, which may be due to the species, multicellular complexity, or our use of a much lower concentration of LPS (50 ng/mL vs 10 µg/mL). Altogether, these data demonstrate that organoids containing APOE3/3 microglia and exposed to a combination of rApoE4 and Aβ (rApoE4 AD model) exhibit clinically relevant pro-inflammatory cytokine production (Figure 5A), GFAP and NfL release (B,C), and microglial lipid accumulation (E).

### 3.2. Stimulus-Based Model of rApoE4 AD

**Figure 6:**
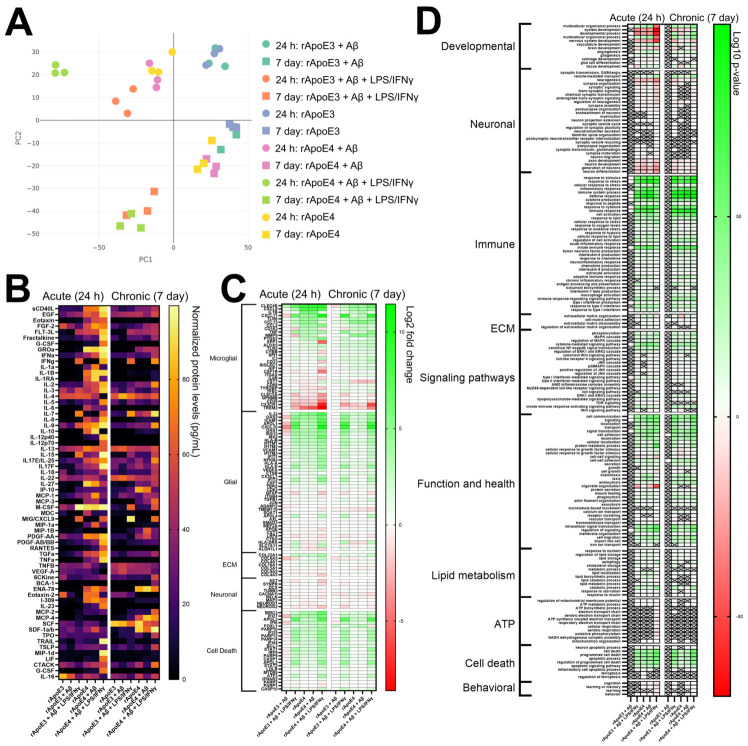
Transcriptomic and proteomic characterization of the rApoE4 AD model using APOE3/3 organoids shows a robust and persistent neuroinflammatory and neurodegenerative phenotype. (**A**) PCA analysis for bulk RNAseq shows clustering of samples between biological replicates indicating high reproducibility of both the model and the stimulation. (**B**) Human cytokine multiplex array was performed using a Luminex panel. For visualization, measurements were normalized to the highest value across sets and measurements below the limit of detection were entered as zero. (**C**) Representative subset of cell-specific genes shows sustained alterations across cell types; significantly differential genes (*p_adj_* ≤ 0.05) identified through bulk RNAseq and visualized in a heatmap colored by Log2 fold change to their respective (24 h or 7-day) rApoE3-treated control. (**D**) Pathway analysis by Gene Ontology enrichment of significantly modulated gene transcriptional changes indicates prolonged and persistent neuroinflammatory and neurodegenerative phenotype; representative subset of significantly modulated pathways, (*p* ≤ 0.05) altered by conditions, visualized in a heatmap colored by Log10 *p*-value to their respective rApoE3 control; only statistically significant (≥1.3) Log10 *p*-values are colored and those that did not reach statistical significance are indicated with an X.

Transcriptomics and supernatant proteomics performed on organoids exposed to rApoE4 acutely (24 h) and chronically (7 days) indicate a strong pro-inflammatory response that is sustained over time (Figure 6).

Transcriptional PCA analysis of the experimental replicates that were assessed by bulk RNA sequencing demonstrates high correlative clustering between the samples (Figure 6A). PCA analysis also indicates that Aβ alone does not induce a strong enough phenotype transcriptionally, as these replicates cluster with control organoids at both time points. In contrast, exposure to pro-inflammatory rApoE4 and/or LPS/IFNγ led to distinct shifting of the experimental replicates, away from controls. Cytokine multiplex analysis at these two time points found that the microglia secrete significant and persistent levels of chemokines and cytokines (Figure 6B; Supplemental Figure S4A,B; Supplemental Tables S1 and S2). Furthermore, additional stimulation with LPS/IFNγ indicates that microglia become sensitized and secrete exacerbated levels of pro-inflammatory elements as compared to those exposed to rApoE3 both acutely (sCD40L, IFNα, IL-2, etc.) and after continuous exposure (IL-2, IL-7, MCP-3, etc.). Transcriptomics of a subset of significantly modulated genes within distinct cell populations, previously identified via scRNAseq, shows acute upregulation of glial and inflammation-related gene expression that is sustained over the course of 7 days (Figure 6C). Pathway analysis similarly shows significant upregulation of immune-related pathways that are sustained and sensitized to LPS/IFNγ stimulation, with enduring downregulation of neuronal-related pathways (Figure 6D). This sensitization to an acute LPS/IFNγ stimulation is a microglial phenotype that has been described in chronic neuroinflammatory conditions of AD and among APOE4/4 carriers [100,101,102].

**Figure 7:**
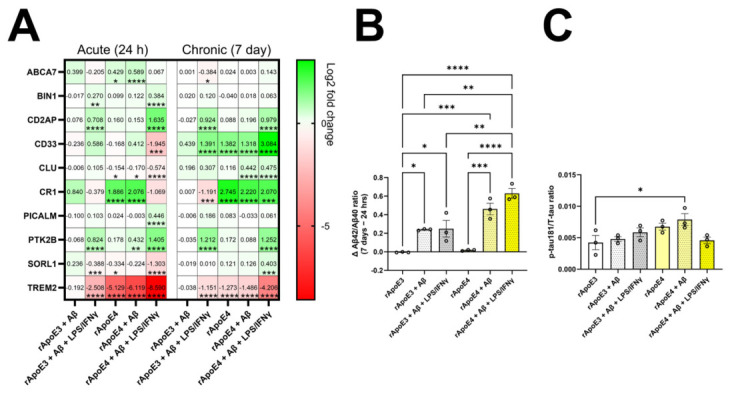
The rApoE4 AD neuroimmune organoid model demonstrates AD-relevant gene expression and increased progression of Aβ42/40 ratio over time and an increased p-tau181/T-tau ratio. (**A**) Transcriptional alterations in GWAS-identified AD risk genes occur in both 24 h and 7-day exposure to rApoE4. Transcriptomics shown were significantly modulated (*p_adj_* ≤ 0.05, values inset as follows: *, *p_adj_* ≤ 0.05; **, *p_adj_* ≤ 0.01; ***, *p_adj_* ≤ 0.001; ****, *p_adj_* ≤ 0.0001) in bulk RNAseq and visualized in a heatmap colored by Log2 fold change (value inset) to their respective (24 h or 7-day) rApoE3-treated control. (**B**) In organoids seeded with Aβ the Aβ42/40 ratio is significantly increased over the course of 7 days in rApoE4-treated organoids. (**C**) The rApoE4 AD model exhibited heightened ratio levels of phosphorylated tau 181 (p-tau181)/total tau (T-tau) after 24 h induction. Levels of tau at the 7-day time point exceeded the range above the standard curve and are not shown. Human amyloid beta and tau measurement were performed using a Luminex panel (3 organoids per condition). Statistics were performed using one-way ANOVA and Tukey’s post hoc test; *p*-value levels of significance are as follows: *, *p* ≤ 0.05; **, *p* ≤ 0.01; ***, *p* ≤ 0.001; ****, *p* ≤ 0.0001.

Differential gene expression of specific GWAS-identified AD risk genes demonstrates modulation of AD-relevant transcripts (Figure 7A) as well as an increased Aβ42/40 ratio in Aβ-seeded organoids exposed to rApoE4 over time (B) and an increased phosphorylated tau 181 over total tau (p-tau181/t-tau) ratio in the rApoE4 AD model after 24 h induction (C). Though both Aβ-seeded organoids treated with rApoE3 and rApoE4 had a significant increase in the Aβ42/40 ratio over the course of 7 days (Supplemental Figure S4C,D), this ratio increased more significantly in rApoE4-treated organoids (Figure 7B). Furthermore, the ratio of p-tau181/t-tau was increased after 24 h (Figure 7C). Unfortunately, for the 7-day time point, measurements of total tau exceeded the limits of detection and ratios could not be quantified in this study. It has been found that ApoE aggregation in microglia leads to seeding of amyloid beta [103] and that ApoE4 increases Aβ [104] and tau [38] pathology relative to other ApoE isoforms. The heightened Aβ42/40 ratio and p-tau181/t-tau in the rApoE4 AD organoids is in agreement with previous observations that AD pathology is exacerbated by chronic neuroinflammation and that levels of inflammation correlate with levels of AD-relevant biomarkers Aβ and tau [105,106,107].

**Figure 8:**
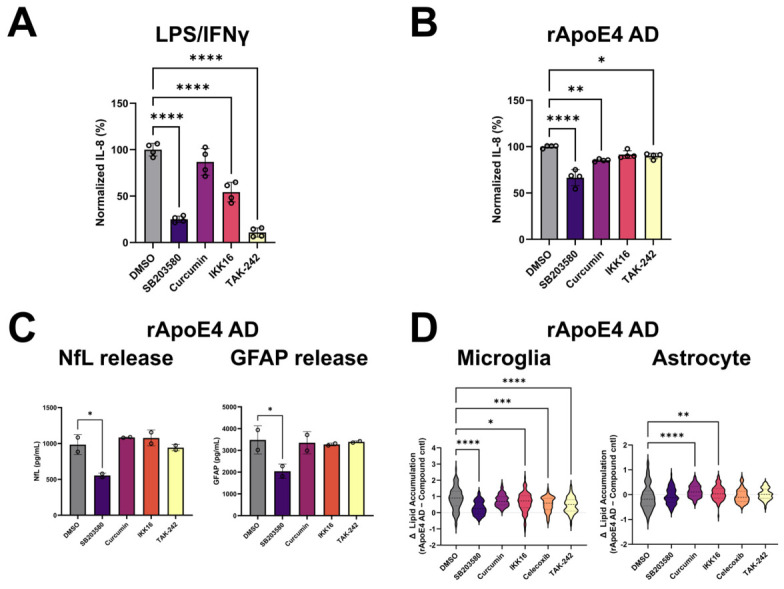
The rApoE4 AD neuroimmune organoid model demonstrates pharmacological pro-inflammatory properties unique to traditional LPS/IFNγ drug screening, with SB203580 (a p38 MAPK inhibitor) exhibiting both anti-inflammatory and neuroprotective properties while reducing microglial lipid accumulation. Treatment with a selection of anti-inflammatory tool compounds on either (**A**) LPS/IFNγ-treated organoids or the (**B**) rApoE4 AD organoids demonstrates adaptability for AD drug screening with readouts for pro-inflammatory microglial activation, (**C**) clinically relevant neuronal and astrocytic biomarkers NfL and GFAP, and (**D**) lipid dysfunction by measurement of cell-specific accumulation. ELISA measurements (2–4 organoids per condition), and volumetric IF measurements from confocal 10X images (*n* = 2 organoids per condition), were performed 3 days after model induction. Organoids were preincubated for 1 h using SB203580 (10 µM), Curcumin (20 µM), IKK16 (1 µM), Celecoxib (10 µM), or TAK-242 (1 µM) prior to induction of rApoE4 AD phenotype. Statistics were performed using one-way ANOVA and Tukey’s post hoc test; *p*-value levels of significance are as follows: *, *p* ≤ 0.05; **, *p* ≤ 0.01; ***, *p* ≤ 0.001; ****, *p* ≤ 0.0001.

Finally, to determine the utility of the rApoE4 AD model in drug development efforts, we tested if an array of known anti-inflammatory compounds could abrogate the neuroinflammatory and neurodegenerative phenotype. We selected compounds based on activated transcriptional pathways and compared our model with an LPS/IFNγ stimulation (Figure 8A,B). Unlike the LPS + IFNγ stimulation, a classic AD drug screening stimulant, the rApoE4 AD stimulation showed substantial (>30%) reduction in chemokine IL-8 only with SB203580, a canonical p38 MAPK inhibitor, while showing minimal response to TAK-242, a TLR4 antagonist. Likewise, only SB203580 reduced both NfL and GFAP release (Figure 8C). Furthermore, lipid staining revealed that SB203580 had the highest reduction (>50%) in rApoE4 AD-induced lipid accumulation within the microglia, without altering lipid levels in the astrocytes (Figure 8D). MAPK signaling has been highly implicated in AD, in chronic inflammation, and in carriers of the APOE4 subtype [108,109,110]. In particular, p38 MAPKs are known to mediate both metabolic dysregulation and pro-inflammatory microglial activity [111,112,113]. These findings demonstrate an exciting opportunity for drug discovery using a novel, highly reproducible human organoid model with an innovative approach to induce phenotypic hallmarks of AD pathogenesis.

## 4. Discussion

Herein, we have described the characterization of a stimulus-based model that induces SLOAD-related pathology through the use of rApoE4 protein in Aβ-seeded organoids. This method yields a robust, reproducible phenotype that exhibits microglial activation and lipid accumulation, and neurodegeneration. Furthermore, we have confirmed that this process can be modulated through pharmaceutical intervention with MAPK inhibitor SB203580. The p38 MAPK pathway is considered a pivotal regulator of inflammation, stimulating pro-apoptotic signaling pathways and excitotoxicity [111,114]. The pathway and its inhibitors have been implicated in a variety of AD-focused drug screening campaigns [109,110,115].

Similar to the use of FAD-related mutations in mouse models and amyloid beta fibrils in in vitro models, the strategy to use recombinant ApoE4 protein in an APOE3/3 organoid is an attempt to model the more prevalent SLOAD. Regardless of the subtype, APOE has been shown to be a critical nexus to the development of AD [29,30]. Preliminary evidence in human AD cortical tissue also demonstrated that intracellular lipid accumulation was present in both APOE3/3 and APOE4/4 carriers, with a slight but not significant increase in APOE4/4 [46], suggesting a similar yet exacerbated or accelerated phenotype in APOE4/4 vs APOE3/3. In our model, we hypothesize that two main mechanisms of action exist in generating the phenotype observed: (1) rApoE4 competitively inhibits the function of endogenous ApoE3 leading to lipid dysfunction and (2) the rApoE4 (full-length or fragments) directly activates the immune response and secretion of pro-inflammatory cytokines. However, we have yet to fully determine the mechanism by which rApoE4 drives neuroinflammation in our model. Due to the differing lipid-binding affinities between ApoE3 and ApoE4, non-lipidated recombinant ApoE was utilized. Non-lipidated ApoE can aggregate which exacerbates Aβ pathology [116,117] with ApoE4 exhibiting enhanced degradation and reduced half-life [118], leading to higher levels of aggregation and Aβ pathology. Furthermore, it has also been shown that ApoE4 fragments are neurotoxic and activate murine BV2 microglia [119,120]. Interestingly, the same study also showed that full-length ApoE4, compared to its toxic fragment, failed to elicit an immune response in murine BV2 microglia despite using 25X the amount used in our current study [120]. This finding may be due to species-specific or model complexity differences.

The results of pathway analysis on the scRNAseq revealed a systems-wide alteration in ATP and mitochondrial-related pathways, caused by a decrease in mitochondrial-related genes. The early stages of AD are marked by mitochondrial dysfunction [121,122,123] believed to be primarily mediated by oxidative stress [124]. High ROS levels promote lipid production and accumulation of toxic peroxidated lipid droplets in glia [125,126]. It is generally believed that microglia dynamically shift from glycolysis and oxidative phosphorylation to meet the energy demands required for homeostatic and reactive processes. During AD progression, the metabolism of microglia shifts from oxidative phosphorylation to aerobic glycolysis [127] and it has been shown that interventions that activate mitochondrial function and facilitate microglial metabolic reprogramming can alleviate neuroinflammation and promote AB clearance [128,129,130]. Though we observed mitochondrial alterations in our model in the scRNAseq, this aspect was not explored in our readouts for compound screening and may provide another phenotype that could be investigated in future studies.

Organoid models have the potential to revolutionize the development of successful therapeutics, with better recapitulation of the native CNS cellular heterogeneity using human-derived cells. Traditional reductionist drug screening does not capture this inherent complexity, and it is unknown whether transfer of conditioned media from these individual monoculture experiments would sufficiently recapitulate the multicellular signaling. Recent initiatives led by the NIH and FDA have emphasized reduction in animal testing and prioritization of human-based research technologies, aiming to advance and improve the translatability of research from bench to bedside. However, validation and qualification of these models is challenging [131] and more work is required by the field to assess the feasibility of performing high-throughput screening on these models as new mechanisms of action may be uncovered in a complex multicellular biological system that have not been previously discovered in a reductionist target-based screen. Furthermore, with recent advances in AI and machine learning [132,133], the laborious and expensive efforts of target deconvolution can be further streamlined to revolutionize the speed and efficiency of pharmacological research in the CNS. Our novel planar organoid system allows for compatibility with high-content imaging systems for live-cell monitoring which would greatly enhance our ability to characterize mechanistic and development processes. Parallel measurements to assess astrocyte and neuronal health, as well as microglial activation, illustrate the ability of a neuroimmune organoid platform to extract multi-factorial, high-content information relevant to human biology for the purposes of CNS drug development and toxicity. As mentioned previously, lipid droplets serve a protective function by protecting against ROS damage. Thus, future characterization of this disease model using lipid-modulating compounds would be of great interest, as many groups have proposed this as a viable target [43,44,45,46,134,135], yet the use of statins for AD has led to conflicting results as to its benefits and harm [136,137,138]. Furthermore, characterization of our AD neuroimmune organoid model using lysosomal- [90], mitochondrial- [121], and metabolic [139,140]-modulating compounds will provide additional mechanistic information. Additionally, the use of genetic approaches, recently described in similar models [141,142], has not been explored and warrants further study.

Finally, an important limitation of our study was the use of a single APOE3/3 donor (male, Caucasian) and APOE4/4 donor (female, Caucasian) in the generation of our organoids. Though these two donors exhibited remarkable similarities in gene transcriptional changes in the scRNAseq, IL-8 secretion, and microglial lipid accumulation, tests in more diverse genetic backgrounds are necessary to prove widespread applicability of the stimulus to induce an AD-like phenotype, such as in APOE2/2 or FAD mutation carriers.

## 5. Conclusions

In summary, we describe a robust stimulus-based AD model that utilizes a combination of rApoE4 protein and Aβ. This stimulus induces sustained pro-inflammatory microglia activation, neurodegeneration, and microglial lipid accumulation which we have characterized over acute (24 h) and chronic (7 days) exposure. Pro-inflammatory IL-8, microglial lipid accumulation, neuronal damage (NfL release) and astrocytic damage (GFAP release) have been assessed as potential pharmacological readouts with high clinical relevance in AD. However, preliminary findings suggest other biomarkers such as the Aβ42/40 ratio, p-tau181/T-tau ratio, or readouts assessing mitochondrial function could be optimized further for future targeted screens. We conclude that this rApoE4 AD neuroimmune organoid model exhibits multiple clinically relevant AD-like phenotypes and allows rapid AD phenotype induction, and robust multicellular readouts from both the collected media and organoid IF staining. Furthermore, the adherent planar morphology of the organoid allows for convenient diffusion of compounds throughout the organoid, as well as in-well IF staining without the need for cryosectioning—major hindrances in the use of spheroidal organoids in large drug screening campaigns. Overall, our model presents a unique opportunity for therapeutic development employing complex multicellular brain organoids, a stimulus-based disease induction that exhibits multiple clinically relevant AD biomarkers, and an organoid morphology that is highly adaptable for high-content screening and drug discovery efforts.

## Figures and Tables

**Table 1:** ScRNAseq sample sets and conditions.

Identity	ID #	Cells	Ave Transcripts Sequenced/Cell	Ave Genes/Cell
APOE3 MG + rApoE4 (24 h)	Acute 1	11,607	28,151	6599
APOE4 MG + rApoE4 (24 h)	Acute 2	22,193	29,207	6726
APOE3 MG + rApoE3 (7 days)	Chronic 1	4772	19,963	6050
APOE3 MG + rApoE3 +Aβ (7 days)	Chronic 2	10,898	19,355	5970
APOE4 MG + rApoE4 (7 days)	Chronic 3	10,251	21,204	6148
APOE4 MG + rApoE4 + Aβ (7 days)	Chronic 4	11,570	21,843	6203

## Data Availability

Omics data sets generated and analyzed during the current study are not publicly available. Reasonable requests for access to specific data may be considered on a case-by-case basis, subject to evaluation of scientific purpose, protection of confidential and proprietary information, and the execution of appropriate confidentiality and data use agreements. Donor consent restrictions require that omics data be made available only through a secure access-controlled database that restricts use to Research Use Only and explicitly prohibits re-identification of the donor or donor relatives and forbids further distribution of the data to any third party. Requests should be directed to the corresponding author.

## Supplementary Materials

The following supporting information can be downloaded at: https://www.mdpi.com/article/10.3390/cells15141266/s1, Supplemental Table S1: ScRNAseq sample sets and conditions; Supplemental Figure S1: Immunofluorescence staining of APOE3/3 or APOE4/4 MG containing organoids treated with combinations of recombinant ApoE and Aβ after chronic (7-day) induction; Supplemental Figure S2: Distribution of cell types detected in the organoid and annotated by scRNAseq; Supplemental Figure S3: Neuroimmune organoids containing either APOE3/3 or APOE4/4 microglia exhibit acute upregulation of IL6; Supplemental Figure S4: AD-relevant IL-6, IL-1β, and Aβ42/40 ratio over time; Supplemental Table S2: Expanded data from proteomic multiplex experiment following acute (24 h) rApoE4 AD induction; Supplemental Table S3: Expanded data from proteomic multiplex experiment following chronic (7-day) rApoE4 AD induction.

## Abbreviations

The following abbreviations are used in this manuscript:

AD	Alzheimer’s Disease
Aβ	Amyloid beta
iPSCs	Induced pluripotent stem cells
EOFAD	Early-onset familial AD
SLOAD	Sporadic late-onset AD
GWAS	Genome-wide association studies
LD	Lipid-droplet
LDAM	LD accumulating microglia
APOE	Apolipoprotein E
NPC	Neural progenitor cell
EC	Endothelial cell
MSC	Mesenchymal stem cell
PEG	Polyethylene glycol
MG	Microglia
NfL	Neurofilament light
GFAP	Glial fibrillary acidic protein
TUJ1	β3 Tubulin
MAPK	Mitogen-activated protein kinase
ROS	Reactive oxygen species
CNS	Central nervous system
NIH	National Institutes of Health
FDA	Food and Drug Administration

## References

[1] Alzheimer’s Association Report (2024). 2024 Alzheimer’s disease facts and figures. Alzheimers Dement..

[2] Manly J.J., Jones R.N., Langa K.M., Ryan L.H., Levine D.A., McCammon R., Heeringa S.G., Weir D. (2022). Estimating the Prevalence of Dementia and Mild Cognitive Impairment in the US: The 2016 Health and Retirement Study Harmonized Cognitive Assessment Protocol Project. JAMA Neurol..

[3] Jonsson L., Wimo A., Handels R., Johansson G., Boada M., Engelborghs S., Frolich L., Jessen F., Kehoe P.G., Kramberger M. (2023). The affordability of lecanemab, an amyloid-targeting therapy for Alzheimer’s disease: An EADC-EC viewpoint. Lancet Reg. Health Eur..

[4] Cummings J.L., Burstein A.H., Fillit H. (2025). Alzheimer Combination Therapies: Overview and Scenarios. J. Prev. Alzheimers Dis..

[5] Drummond E., Wisniewski T. (2017). Alzheimer’s disease: Experimental models and reality. Acta Neuropathol..

[6] Scheltens P., De Strooper B., Kivipelto M., Holstege H., Chetelat G., Teunissen C.E., Cummings J., van der Flier W.M. (2021). Alzheimer’s disease. Lancet.

[7] Efthymiou A.G., Goate A.M. (2017). Late onset Alzheimer’s disease genetics implicates microglial pathways in disease risk. Mol. Neurodegener..

[8] Shi F.D., Yong V.W. (2025). Neuroinflammation across neurological diseases. Science.

[9] Fruhwurth S., Zetterberg H., Paludan S.R. (2024). Microglia and amyloid plaque formation in Alzheimer’s disease—Evidence, possible mechanisms, and future challenges. J. Neuroimmunol..

[10] Baligacs N., Albertini G., Borrie S.C., Serneels L., Pridans C., Balusu S., De Strooper B. (2024). Homeostatic microglia initially seed and activated microglia later reshape amyloid plaques in Alzheimer’s Disease. Nat. Commun..

[11] Foley P. (2010). Lipids in Alzheimer’s disease: A century-old story. Biochim. Biophys. Acta.

[12] Tong B., Ba Y., Li Z., Yang C., Su K., Qi H., Zhang D., Liu X., Wu Y., Chen Y. (2024). Targeting dysregulated lipid metabolism for the treatment of Alzheimer’s disease and Parkinson’s disease: Current advancements and future prospects. Neurobiol. Dis..

[13] Marschallinger J., Iram T., Zardeneta M., Lee S.E., Lehallier B., Haney M.S., Pluvinage J.V., Mathur V., Hahn O., Morgens D.W. (2020). Author Correction: Lipid-droplet-accumulating microglia represent a dysfunctional and proinflammatory state in the aging brain. Nat. Neurosci..

[14] Yang D., Wang X., Zhang L., Fang Y., Zheng Q., Liu X., Yu W., Chen S., Ying J., Hua F. (2022). Lipid metabolism and storage in neuroglia: Role in brain development and neurodegenerative diseases. Cell Biosci..

[15] Wang L., Qu F., Yu X., Yang S., Zhao B., Chen Y., Li P., Zhang Z., Zhang J., Han X. (2024). Cortical lipid metabolic pathway alteration of early Alzheimer’s disease and candidate drugs screen. Eur. J. Med. Res..

[16] Yassine H.N., Croteau E., Rawat V., Hibbeln J.R., Rapoport S.I., Cunnane S.C., Umhau J.C. (2017). DHA brain uptake and APOE4 status: A PET study with [1-(11)C]-DHA. Alzheimers Res. Ther..

[17] Ooi K.M., Vacy K., Boon W.C. (2021). Fatty acids and beyond: Age and Alzheimer’s disease related changes in lipids reveal the neuro-nutraceutical potential of lipids in cognition. Neurochem. Int..

[18] Zhao X., Zhang S., Sanders A.R., Duan J. (2023). Brain Lipids and Lipid Droplet Dysregulation in Alzheimer’s Disease and Neuropsychiatric Disorders. Complex Psychiatry.

[19] He S., Xu Z., Han X. (2025). Lipidome disruption in Alzheimer’s disease brain: Detection, pathological mechanisms, and therapeutic implications. Mol. Neurodegener..

[20] Agarwal M., Khan S. (2020). Plasma Lipids as Biomarkers for Alzheimer’s Disease: A Systematic Review. Cureus.

[21] Liu Y., Thalamuthu A., Mather K.A., Crawford J., Ulanova M., Wong M.W.K., Pickford R., Sachdev P.S., Braidy N. (2021). Plasma lipidome is dysregulated in Alzheimer’s disease and is associated with disease risk genes. Transl. Psychiatry.

[22] Liu C.C., Kanekiyo T., Xu H., Bu G. (2013). Correction: Apolipoprotein E and Alzheimer disease: Risk, mechanisms and therapy. Nat. Rev. Neurol..

[23] van der Lee S.J., Wolters F.J., Ikram M.K., Hofman A., Ikram M.A., Amin N., van Duijn C.M. (2018). Correction: The effect of APOE and other common genetic variants on the onset of Alzheimer’s disease and dementia: A community-based cohort study. Lancet Neurol..

[24] Corder E.H., Saunders A.M., Strittmatter W.J., Schmechel D.E., Gaskell P.C., Small G.W., Roses A.D., Haines J.L., Pericak-Vance M.A. (1993). Gene dose of apolipoprotein E type 4 allele and the risk of Alzheimer’s disease in late onset families. Science.

[25] Saunders A.M., Strittmatter W.J., Schmechel D., George-Hyslop P.H., Pericak-Vance M.A., Joo S.H., Rosi B.L., Gusella J.F., Crapper-MacLachlan D.R., Alberts M.J. (1993). Association of apolipoprotein E allele epsilon 4 with late-onset familial and sporadic Alzheimer’s disease. Neurology.

[26] Millet A., Ledo J.H., Tavazoie S.F. (2024). An exhausted-like microglial population accumulates in aged and APOE4 genotype Alzheimer’s brains. Immunity.

[27] Liu C.C., Zhao N., Fu Y., Wang N., Linares C., Tsai C.W., Bu G. (2017). ApoE4 Accelerates Early Seeding of Amyloid Pathology. Neuron.

[28] Essenburg C., Ramanathan M. (2025). APOE4 genotypes and the trajectory of biomarkers, neuroimaging, and cognitive measures in Alzheimer’s Disease: A mixed-effects disease progression model. J. Pharmacokinet. Pharmacodyn..

[29] Liu T., Zhu B., Liu Y., Zhang X., Yin J., Li X., Jiang L., Hodges A.P., Rosenthal S.B., Zhou L. (2020). Multi-omic comparison of Alzheimer’s variants in human ESC-derived microglia reveals convergence at APOE. J. Exp. Med..

[30] Williams D.M., Heikkinen S., Hiltunen M., FinnGen, Davies N.M., Anderson E.L. (2026). The proportion of Alzheimer’s disease attributable to apolipoprotein E. npj Dement..

[31] Fernandez-Calle R., Konings S.C., Frontinan-Rubio J., Garcia-Revilla J., Camprubi-Ferrer L., Svensson M., Martinson I., Boza-Serrano A., Venero J.L., Nielsen H.M. (2022). APOE in the bullseye of neurodegenerative diseases: Impact of the APOE genotype in Alzheimer’s disease pathology and brain diseases. Mol. Neurodegener..

[32] Pitas R.E., Boyles J.K., Lee S.H., Foss D., Mahley R.W. (1987). Astrocytes synthesize apolipoprotein E and metabolize apolipoprotein E-containing lipoproteins. Biochim. Biophys. Acta.

[33] Blumenfeld J., Yip O., Kim M.J., Huang Y. (2024). Cell type-specific roles of APOE4 in Alzheimer disease. Nat. Rev. Neurosci..

[34] Krasemann S., Madore C., Cialic R., Baufeld C., Calcagno N., El Fatimy R., Beckers L., O’Loughlin E., Xu Y., Fanek Z. (2017). The TREM2-APOE Pathway Drives the Transcriptional Phenotype of Dysfunctional Microglia in Neurodegenerative Diseases. Immunity.

[35] Preman P., Moechars D., Fertan E., Wolfs L., Serneels L., Shah D., Lamote J., Poovathingal S., Snellinx A., Mancuso R. (2024). APOE from astrocytes restores Alzheimer’s Abeta-pathology and DAM-like responses in APOE deficient microglia. EMBO Mol. Med..

[36] Qi G., Mi Y., Shi X., Gu H., Brinton R.D., Yin F. (2021). ApoE4 Impairs Neuron-Astrocyte Coupling of Fatty Acid Metabolism. Cell Rep..

[37] Victor M.B., Leary N., Luna X., Meharena H.S., Scannail A.N., Bozzelli P.L., Samaan G., Murdock M.H., von Maydell D., Effenberger A.H. (2022). Lipid accumulation induced by APOE4 impairs microglial surveillance of neuronal-network activity. Cell Stem Cell.

[38] Shi Y., Yamada K., Liddelow S.A., Smith S.T., Zhao L., Luo W., Tsai R.M., Spina S., Grinberg L.T., Rojas J.C. (2017). ApoE4 markedly exacerbates tau-mediated neurodegeneration in a mouse model of tauopathy. Nature.

[39] Ferrari-Souza J.P., Lussier F.Z., Leffa D.T., Therriault J., Tissot C., Bellaver B., Ferreira P.C.L., Malpetti M., Wang Y.T., Povala G. (2023). APOEepsilon4 associates with microglial activation independently of Abeta plaques and tau tangles. Sci. Adv..

[40] Lin Y.T., Seo J., Gao F., Feldman H.M., Wen H.L., Penney J., Cam H.P., Gjoneska E., Raja W.K., Cheng J. (2018). APOE4 Causes Widespread Molecular and Cellular Alterations Associated with Alzheimer’s Disease Phenotypes in Human iPSC-Derived Brain Cell Types. Neuron.

[41] Liu L., MacKenzie K.R., Putluri N., Maletic-Savatic M., Bellen H.J. (2017). The Glia-Neuron Lactate Shuttle and Elevated ROS Promote Lipid Synthesis in Neurons and Lipid Droplet Accumulation in Glia via APOE/D. Cell Metab..

[42] Moulton M.J., Barish S., Ralhan I., Chang J., Goodman L.D., Harland J.G., Marcogliese P.C., Johansson J.O., Ioannou M.S., Bellen H.J. (2021). Neuronal ROS-induced glial lipid droplet formation is altered by loss of Alzheimer’s disease-associated genes. Proc. Natl. Acad. Sci. USA.

[43] Prakash P., Manchanda P., Paouri E., Bisht K., Sharma K., Rajpoot J., Wendt V., Hossain A., Wijewardhane P.R., Randolph C.E. (2025). Amyloid-beta induces lipid droplet-mediated microglial dysfunction via the enzyme DGAT2 in Alzheimer’s disease. Immunity.

[44] Friday C.M., Stephens I.O., Smith C.T., Lee S., Satish D., Devanney N.A., Cohen S., Morganti J.M., Gordon S.M., Johnson L.A. (2025). APOE4 reshapes the lipid droplet proteome and modulates microglial inflammatory responses. Neurobiol. Dis..

[45] Huynh T.N., Fikse E.N., De La Torre A.L., Havrda M.C., Chang C.C.Y., Chang T.Y. (2024). Inhibiting the Cholesterol Storage Enzyme ACAT1/SOAT1 in Aging Apolipoprotein E4 Mice Alters Their Brains’ Inflammatory Profiles. Int. J. Mol. Sci..

[46] Haney M.S., Palovics R., Munson C.N., Long C., Johansson P.K., Yip O., Dong W., Rawat E., West E., Schlachetzki J.C.M. (2024). APOE4/4 is linked to damaging lipid droplets in Alzheimer’s disease microglia. Nature.

[47] Gosselin D., Skola D., Coufal N.G., Holtman I.R., Schlachetzki J.C.M., Sajti E., Jaeger B.N., O’Connor C., Fitzpatrick C., Pasillas M.P. (2017). An environment-dependent transcriptional network specifies human microglia identity. Science.

[48] McKean N.E., Handley R.R., Snell R.G. (2021). A Review of the Current Mammalian Models of Alzheimer’s Disease and Challenges That Need to Be Overcome. Int. J. Mol. Sci..

[49] Geirsdottir L., David E., Keren-Shaul H., Weiner A., Bohlen S.C., Neuber J., Balic A., Giladi A., Sheban F., Dutertre C.A. (2019). Cross-Species Single-Cell Analysis Reveals Divergence of the Primate Microglia Program. Cell.

[50] Li J., Pan L., Pembroke W.G., Rexach J.E., Godoy M.I., Condro M.C., Alvarado A.G., Harteni M., Chen Y.W., Stiles L. (2021). Conservation and divergence of vulnerability and responses to stressors between human and mouse astrocytes. Nat. Commun..

[51] Linnerbauer M., Wheeler M.A., Quintana F.J. (2020). Astrocyte Crosstalk in CNS Inflammation. Neuron.

[52] Ding Z., Guo S., Luo L., Zheng Y., Gan S., Kang X., Wu X., Zhu S. (2021). Emerging Roles of Microglia in Neuro-vascular Unit: Implications of Microglia-Neurons Interactions. Front. Cell Neurosci..

[53] Qian X., Song H., Ming G.L. (2019). Brain organoids: Advances, applications and challenges. Development.

[54] Zhang W., Jiang J., Xu Z., Yan H., Tang B., Liu C., Chen C., Meng Q. (2023). Microglia-containing human brain organoids for the study of brain development and pathology. Mol. Psychiatry.

[55] Yuan N.Y., Richards W.D., Parham K.T., Clark S.G., Greuel K., Polzin B., Smith S.W., Lebakken C.S. (2025). Neural organoids incorporating microglia to assess neuroinflammation and toxicities induced by known developmental neurotoxins. Curr. Res. Toxicol..

[56] Ji Y., Chen X., Wang Z., Meek C.J., McLean J.L., Yang Y., Yuan C., Rochet J.C., Liu F., Xu R. (2025). Alzheimer’s disease patient brain extracts induce multiple pathologies in novel vascularized neuroimmune organoids for disease modeling and drug discovery. Mol. Psychiatry.

[57] Chen X., Sun G., Tian E., Zhang M., Davtyan H., Beach T.G., Reiman E.M., Blurton-Jones M., Holtzman D.M., Shi Y. (2021). Modeling Sporadic Alzheimer’s Disease in Human Brain Organoids under Serum Exposure. Adv. Sci..

[58] Gonzalez C., Armijo E., Bravo-Alegria J., Becerra-Calixto A., Mays C.E., Soto C. (2018). Modeling amyloid beta and tau pathology in human cerebral organoids. Mol. Psychiatry.

[59] Kwak S.S., Washicosky K.J., Brand E., von Maydell D., Aronson J., Kim S., Capen D.E., Cetinbas M., Sadreyev R., Ning S. (2020). Amyloid-beta42/40 ratio drives tau pathology in 3D human neural cell culture models of Alzheimer’s disease. Nat. Commun..

[60] Meyer-Acosta K.K., Diaz-Guerra E., Varma P., Aruk A., Mirsadeghi S., Muniz-Perez A., Rafati Y., Hosseini A., Nieto-Estevez V., Giugliano M. (2025). APOE4 impacts cortical neurodevelopment and alters network formation in human brain organoids. Stem Cell Rep..

[61] Barry C., Schmitz M.T., Propson N.E., Hou Z., Zhang J., Nguyen B.K., Bolin J.M., Jiang P., McIntosh B.E., Probasco M.D. (2017). Uniform neural tissue models produced on synthetic hydrogels using standard culture techniques. Exp. Biol. Med..

[62] Majumder J., Torr E.E., Aisenbrey E.A., Lebakken C.S., Favreau P.F., Richards W.D., Yin Y., Chang Q., Murphy W.L. (2024). Human induced pluripotent stem cell-derived planar neural organoids assembled on synthetic hydrogels. J. Tissue Eng..

[63] Schwartz M.P., Hou Z., Propson N.E., Zhang J., Engstrom C.J., Santos Costa V., Jiang P., Nguyen B.K., Bolin J.M., Daly W. (2015). Human pluripotent stem cell-derived neural constructs for predicting neural toxicity. Proc. Natl. Acad. Sci. USA.

[64] Schindelin J., Arganda-Carreras I., Frise E., Kaynig V., Longair M., Pietzsch T., Preibisch S., Rueden C., Saalfeld S., Schmid B. (2012). Fiji: An open-source platform for biological-image analysis. Nat. Methods.

[65] Ollion J., Cochennec J., Loll F., Escude C., Boudier T. (2013). TANGO: A generic tool for high-throughput 3D image analysis for studying nuclear organization. Bioinformatics.

[66] Hao Y., Stuart T., Kowalski M.H., Choudhary S., Hoffman P., Hartman A., Srivastava A., Molla G., Madad S., Fernandez-Granda C. (2024). Dictionary learning for integrative, multimodal and scalable single-cell analysis. Nat. Biotechnol..

[67] Subramanian A., Tamayo P., Mootha V.K., Mukherjee S., Ebert B.L., Gillette M.A., Paulovich A., Pomeroy S.L., Golub T.R., Lander E.S. (2005). Gene set enrichment analysis: A knowledge-based approach for interpreting genome-wide expression profiles. Proc. Natl. Acad. Sci. USA.

[68] Chicco D., Agapito G. (2022). Nine quick tips for pathway enrichment analysis. PLoS Comput. Biol..

[69] Kolberg L., Raudvere U., Kuzmin I., Vilo J., Peterson H. (2020). gprofiler2—An R package for gene list functional enrichment analysis and namespace conversion toolset g:Profiler. F1000Research.

[70] Sievert C. (2020). Interactive Web-Based Data Visualization with R, Plotly and Shiny.

[71] Zhang Y., Sloan S.A., Clarke L.E., Caneda C., Plaza C.A., Blumenthal P.D., Vogel H., Steinberg G.K., Edwards M.S., Li G. (2016). Purification and Characterization of Progenitor and Mature Human Astrocytes Reveals Transcriptional and Functional Differences with Mouse. Neuron.

[72] Lanfranco M.F., Sepulveda J., Kopetsky G., Rebeck G.W. (2021). Expression and secretion of apoE isoforms in astrocytes and microglia during inflammation. Glia.

[73] Yang L.G., March Z.M., Stephenson R.A., Narayan P.S. (2023). Apolipoprotein E in lipid metabolism and neurodegenerative disease. Trends Endocrinol. Metab..

[74] Arnaud L., Benech P., Greetham L., Stephan D., Jimenez A., Jullien N., Garcia-Gonzalez L., Tsvetkov P.O., Devred F., Sancho-Martinez I. (2022). APOE4 drives inflammation in human astrocytes via TAGLN3 repression and NF-kappaB activation. Cell Rep..

[75] Ramakrishna S., Jhaveri V., Konings S.C., Nawalpuri B., Chakraborty S., Holst B., Schmid B., Gouras G.K., Freude K.K., Muddashetty R.S. (2021). APOE4 Affects Basal and NMDAR-Mediated Protein Synthesis in Neurons by Perturbing Calcium Homeostasis. J. Neurosci..

[76] Wang C., Lu J., Sha X., Qiu Y., Chen H., Yu Z. (2023). TRPV1 regulates ApoE4-disrupted intracellular lipid homeostasis and decreases synaptic phagocytosis by microglia. Exp. Mol. Med..

[77] Lanfranco M.F., Ng C.A., Rebeck G.W. (2020). ApoE Lipidation as a Therapeutic Target in Alzheimer’s Disease. Int. J. Mol. Sci..

[78] Huebbe P., Rimbach G. (2017). Evolution of human apolipoprotein E (APOE) isoforms: Gene structure, protein function and interaction with dietary factors. Ageing Res. Rev..

[79] Chen J., Li Q., Wang J. (2011). Topology of human apolipoprotein E3 uniquely regulates its diverse biological functions. Proc. Natl. Acad. Sci. USA.

[80] Grimaldi L., Bovi E., Formisano R., Sancesario G. (2024). ApoE: The Non-Protagonist Actor in Neurological Diseases. Genes.

[81] Capogna E., Watne L.O., Sorensen O., Guichelaar C.J., Idland A.V., Halaas N.B., Blennow K., Zetterberg H., Walhovd K.B., Fjell A.M. (2023). Associations of neuroinflammatory IL-6 and IL-8 with brain atrophy, memory decline, and core AD biomarkers—In cognitively unimpaired older adults. Brain Behav. Immun..

[82] Galimberti D., Schoonenboom N., Scheltens P., Fenoglio C., Bouwman F., Venturelli E., Guidi I., Blankenstein M.A., Bresolin N., Scarpini E. (2006). Intrathecal chemokine synthesis in mild cognitive impairment and Alzheimer disease. Arch. Neurol..

[83] Taipa R., das Neves S.P., Sousa A.L., Fernandes J., Pinto C., Correia A.P., Santos E., Pinto P.S., Carneiro P., Costa P. (2019). Proinflammatory and anti-inflammatory cytokines in the CSF of patients with Alzheimer’s disease and their correlation with cognitive decline. Neurobiol. Aging.

[84] Franco-Bocanegra D.K., Gourari Y., McAuley C., Chatelet D.S., Johnston D.A., Nicoll J.A.R., Boche D. (2021). Microglial morphology in Alzheimer’s disease and after Abeta immunotherapy. Sci. Rep..

[85] Dias D., Portugal C.C., Relvas J., Socodato R. (2025). From Genetics to Neuroinflammation: The Impact of ApoE4 on Microglial Function in Alzheimer’s Disease. Cells.

[86] Montagne A., Nikolakopoulou A.M., Huuskonen M.T., Sagare A.P., Lawson E.J., Lazic D., Rege S.V., Grond A., Zuniga E., Barnes S.R. (2021). APOE4 accelerates advanced-stage vascular and neurodegenerative disorder in old Alzheimer’s mice via cyclophilin A independently of amyloid-beta. Nat. Aging.

[87] Tai L.M., Thomas R., Marottoli F.M., Koster K.P., Kanekiyo T., Morris A.W., Bu G. (2016). The role of APOE in cerebrovascular dysfunction. Acta Neuropathol..

[88] Chen J.X., Yan S.D. (2007). Amyloid-beta-induced mitochondrial dysfunction. J. Alzheimers Dis..

[89] Lee S., Williams H.C., Gorman A.A., Devanney N.A., Harrison D.A., Walsh A.E., Goulding D.S., Tuck T., Schwartz J.L., Zajac D.J. (2023). APOE4 drives transcriptional heterogeneity and maladaptive immunometabolic responses of astrocytes. bioRxiv.

[90] Hellen M., Weert I., Muller S.A., Rasanen N., Kettunen P., Lehtonen S., Peitz M., Fliessbach K., Takalo M., Koskuvi M. (2025). Inflammation-induced lysosomal dysfunction in human iPSC-derived microglia is exacerbated by APOE 4/4 genotype. J. Neuroinflamm..

[91] Lee S., Devanney N.A., Golden L.R., Smith C.T., Schwartz J.L., Walsh A.E., Clarke H.A., Goulding D.S., Allenger E.J., Morillo-Segovia G. (2023). APOE modulates microglial immunometabolism in response to age, amyloid pathology, and inflammatory challenge. Cell Rep..

[92] Nabilah N., Ahmad Puzi N.N., Vidyadaran S. (2020). Microglia-induced Neurotoxicity: A Review of in Vitro Co-culture Models. Malays. J. Med. Health Sci..

[93] Volpato V., Webber C. (2020). Addressing variability in iPSC-derived models of human disease: Guidelines to promote reproducibility. Dis. Models Mech..

[94] Carcamo-Orive I., Hoffman G.E., Cundiff P., Beckmann N.D., D’Souza S.L., Knowles J.W., Patel A., Papatsenko D., Abbasi F., Reaven G.M. (2017). Analysis of Transcriptional Variability in a Large Human iPSC Library Reveals Genetic and Non-genetic Determinants of Heterogeneity. Cell Stem Cell.

[95] Mielke M.M., Syrjanen J.A., Blennow K., Zetterberg H., Vemuri P., Skoog I., Machulda M.M., Kremers W.K., Knopman D.S., Jack C. (2019). Plasma and CSF neurofilament light: Relation to longitudinal neuroimaging and cognitive measures. Neurology.

[96] Fuloria N.K., Sekar M., Porwal O., Ansari M.T., Biswas A., Narain K., Biswas S., Bhatia S., Dhanalekshmi U.M., Fuloria S. (2026). Neurofilament light chain in Alzheimer’s disease. Clin. Chim. Acta.

[97] Zou Y., Wang Y., Ma X., Mu D., Zhong J., Ma C., Mao C., Yu S., Gao J., Qiu L. (2024). CSF and blood glial fibrillary acidic protein for the diagnosis of Alzheimer’s disease: A systematic review and meta-analysis. Ageing Res. Rev..

[98] Ghanbarian E., Qian T., Khorsand B., Zheng L., Sajjadi S.A., Grill J.D., Rabin L.A., Lipton R.B., Sperling R.A., Ezzati A. (2025). Prognostic value of plasma glial fibrillary acidic protein in cognitively unimpaired older adults: Results from the A4 study. Alzheimers Dement..

[99] Khatchadourian A., Bourque S.D., Richard V.R., Titorenko V.I., Maysinger D. (2012). Dynamics and regulation of lipid droplet formation in lipopolysaccharide (LPS)-stimulated microglia. Biochim. Biophys. Acta.

[100] Li J.W., Zong Y., Cao X.P., Tan L., Tan L. (2018). Microglial priming in Alzheimer’s disease. Ann. Transl. Med..

[101] Bivona G., Iemmolo M., Agnello L., Lo Sasso B., Gambino C.M., Giglio R.V., Scazzone C., Ghersi G., Ciaccio M. (2023). Microglial Activation and Priming in Alzheimer’s Disease: State of the Art and Future Perspectives. Int. J. Mol. Sci..

[102] Liu A., Wang T., Yang L., Zhou Y. (2025). The APOE-Microglia Axis in Alzheimer’s Disease: Functional Divergence and Therapeutic Perspectives-A Narrative Review. Brain Sci..

[103] Kaji S., Berghoff S.A., Spieth L., Schlaphoff L., Sasmita A.O., Vitale S., Buschgens L., Kedia S., Zirngibl M., Nazarenko T. (2024). Apolipoprotein E aggregation in microglia initiates Alzheimer’s disease pathology by seeding beta-amyloidosis. Immunity.

[104] Holtzman D.M., Herz J., Bu G. (2012). Apolipoprotein E and apolipoprotein E receptors: Normal biology and roles in Alzheimer disease. Cold Spring Harb. Perspect. Med..

[105] Minter M.R., Taylor J.M., Crack P.J. (2016). The contribution of neuroinflammation to amyloid toxicity in Alzheimer’s disease. J. Neurochem..

[106] Ebrahimi R., Bordbar S., Azad G., Davoody S., Mahmoudi M., Esmaeilpour K. (2025). Beyond Neuroinflammation: Microglia at the Crossroads of Amyloid, Tau, and Neurodegeneration in Alzheimer’s Disease. Neurol. Sci..

[107] Ismail R., Parbo P., Madsen L.S., Hansen A.K., Hansen K.V., Schaldemose J.L., Kjeldsen P.L., Stokholm M.G., Gottrup H., Eskildsen S.F. (2020). The relationships between neuroinflammation, beta-amyloid and tau deposition in Alzheimer’s disease: A longitudinal PET study. J. Neuroinflamm..

[108] Yang F., Zhao L.J., Xu Q., Zhao J. (2024). The journey of p38 MAP kinase inhibitors: From bench to bedside in treating inflammatory diseases. Eur. J. Med. Chem..

[109] Park J.C., Jang S.Y., Lee D., Lee J., Kang U., Chang H., Kim H.J., Han S.H., Seo J., Choi M. (2021). A logical network-based drug-screening platform for Alzheimer’s disease representing pathological features of human brain organoids. Nat. Commun..

[110] Naderi Yeganeh P., Kwak S.S., Jorfi M., Koler K., Kalatturu T., von Maydell D., Liu Z., Guo K., Choi Y., Park J. (2025). Integrative pathway analysis across humans and 3D cellular models identifies the p38 MAPK-MK2 axis as a therapeutic target for Alzheimer’s disease. Neuron.

[111] Bachstetter A.D., Xing B., de Almeida L., Dimayuga E.R., Watterson D.M., Van Eldik L.J. (2011). Microglial p38alpha MAPK is a key regulator of proinflammatory cytokine up-regulation induced by toll-like receptor (TLR) ligands or beta-amyloid (Abeta). J. Neuroinflamm..

[112] He Y., She H., Zhang T., Xu H., Cheng L., Yepes M., Zhao Y., Mao Z. (2018). p38 MAPK inhibits autophagy and promotes microglial inflammatory responses by phosphorylating ULK1. J. Cell Biol..

[113] Ma X., Zhang Y., Gou D., Ma J., Du J., Wang C., Li S., Cui H. (2022). Metabolic Reprogramming of Microglia Enhances Proinflammatory Cytokine Release through EphA2/p38 MAPK Pathway in Alzheimer’s Disease. J. Alzheimers Dis..

[114] Rivera-Cervantes M.C., Castaneda-Arellano R., Castro-Torres R.D., Gudino-Cabrera G., Feria y Velasco A.I., Camins A., Beas-Zarate C. (2015). P38 MAPK inhibition protects against glutamate neurotoxicity and modifies NMDA and AMPA receptor subunit expression. J. Mol. Neurosci..

[115] Birkle T.J.Y., Willems H.M.G., Skidmore J., Brown G.C. (2024). Disease phenotypic screening in neuron-glia cocultures identifies blockers of inflammatory neurodegeneration. iScience.

[116] Liu C.C., Murray M.E., Li X., Zhao N., Wang N., Heckman M.G., Shue F., Martens Y., Li Y., Raulin A.C. (2021). APOE3-Jacksonville (V236E) variant reduces self-aggregation and risk of dementia. Sci. Transl. Med..

[117] Hatters D.M., Zhong N., Rutenber E., Weisgraber K.H. (2006). Amino-terminal domain stability mediates apolipoprotein E aggregation into neurotoxic fibrils. J. Mol. Biol..

[118] Riddell D.R., Zhou H., Atchison K., Warwick H.K., Atkinson P.J., Jefferson J., Xu L., Aschmies S., Kirksey Y., Hu Y. (2008). Impact of apolipoprotein E (ApoE) polymorphism on brain ApoE levels. J. Neurosci..

[119] Orr A.L., Kim C., Jimenez-Morales D., Newton B.W., Johnson J.R., Krogan N.J., Swaney D.L., Mahley R.W. (2019). Neuronal Apolipoprotein E4 Expression Results in Proteome-Wide Alterations and Compromises Bioenergetic Capacity by Disrupting Mitochondrial Function. J. Alzheimers Dis..

[120] Rohn T.T., Beck J.D., Galla S.J., Isho N.F., Pollock T.B., Suresh T., Kulkarni A., Sanghal T., Hayden E.J. (2021). Fragmentation of Apolipoprotein E4 is Required for Differential Expression of Inflammation and Activation Related Genes in Microglia Cells. Int. J. Neurodegener. Dis..

[121] McGill Percy K.C., Liu Z., Qi X. (2025). Mitochondrial dysfunction in Alzheimer’s disease: Guiding the path to targeted therapies. Neurotherapeutics.

[122] Reddy P.H., Tripathi R., Troung Q., Tirumala K., Reddy T.P., Anekonda V., Shirendeb U.P., Calkins M.J., Reddy A.P., Mao P. (2012). Abnormal mitochondrial dynamics and synaptic degeneration as early events in Alzheimer’s disease: Implications to mitochondria-targeted antioxidant therapeutics. Biochim. Biophys. Acta.

[123] Pradeepkiran J.A., Reddy P.H. (2020). Defective mitophagy in Alzheimer’s disease. Ageing Res. Rev..

[124] Wang W., Zhao F., Ma X., Perry G., Zhu X. (2020). Mitochondria dysfunction in the pathogenesis of Alzheimer’s disease: Recent advances. Mol. Neurodegener..

[125] Goodman L.D., Ralhan I., Li X., Lu S., Moulton M.J., Park Y.J., Zhao P., Kanca O., Ghaderpour Taleghani Z.S., Jacquemyn J. (2024). Tau is required for glial lipid droplet formation and resistance to neuronal oxidative stress. Nat. Neurosci..

[126] Lee S.J., Zhang J., Choi A.M., Kim H.P. (2013). Mitochondrial dysfunction induces formation of lipid droplets as a generalized response to stress. Oxid. Med. Cell. Longev..

[127] Baik S.H., Kang S., Lee W., Choi H., Chung S., Kim J.I., Mook-Jung I. (2019). A Breakdown in Metabolic Reprogramming Causes Microglia Dysfunction in Alzheimer’s Disease. Cell Metab..

[128] Song G.J., Suk K. (2017). Pharmacological Modulation of Functional Phenotypes of Microglia in Neurodegenerative Diseases. Front. Aging Neurosci..

[129] Qi G., Mi Y., Yin F. (2019). Cellular Specificity and Inter-cellular Coordination in the Brain Bioenergetic System: Implications for Aging and Neurodegeneration. Front. Physiol..

[130] Wang Y., Huang Y., Xu Y., Ruan W., Wang H., Zhang Y., Saavedra J.M., Zhang L., Huang Z., Pang T. (2018). Correction to: A Dual AMPK/Nrf2 Activator Reduces Brain Inflammation After Stroke by Enhancing Microglia M2 Polarization. Antioxid. Redox Signal.

[131] Wang D., Villenave R., Stokar-Regenscheit N., Clevers H. (2025). Human organoids as 3D in vitro platforms for drug discovery: Opportunities and challenges. Nat. Rev. Drug Discov..

[132] Ravichandran R., Park S., Skorupan S., Bessette K., Gentile F., Yu B., Zhan P. (2025). Chapter 1—Artificial intelligence in early stages of structure-based drug discovery. Drug Discovery Stories.

[133] Wang X., Zhang M., Xu J., Li X., Xiong J., Cao H., Dou F., Zhai X., Sun H. (2025). A novel approach for target deconvolution from phenotype-based screening using knowledge graph. Sci. Rep..

[134] Meier S., Larsen A.S.G., Wanke F., Mercado N., Mei A., Takacs L., Mracsko E.S., Collin L., Kampmann M., Roudnicky F. (2025). An efficient, non-viral arrayed CRISPR screening platform for iPSC-derived myeloid and microglia models. Stem Cell Rep..

[135] Stephenson R.A., Sepulveda J., Johnson K.R., Lita A., Gopalakrishnan J., Acri D.J., Beilina A., Cheng L., Yang L.G., Root J.T. (2025). Triglyceride metabolism controls inflammation and microglial phenotypes associated with APOE4. Cell Rep..

[136] Westphal Filho F.L., Moss Lopes P.R., Menegaz de Almeida A., Sano V.K.T., Tamashiro F.M., Goncalves O.R., de Moraes F.C.A., Kreuz M., Kelly F.A., Silveira Feitoza P.V. (2025). Statin use and dementia risk: A systematic review and updated meta-analysis. Alzheimers Dement..

[137] Ye Z., Deng J., Wu X., Cai J., Li S., Chen X., Xin J. (2025). Association of statins use and genetic susceptibility with incidence of Alzheimer’s disease. J. Prev. Alzheimers Dis..

[138] Zhou Z., Ryan J., Ernst M.E., Zoungas S., Tonkin A.M., Woods R.L., McNeil J.J., Reid C.M., Curtis A.J., Wolfe R. (2021). Effect of Statin Therapy on Cognitive Decline and Incident Dementia in Older Adults. J. Am. Coll. Cardiol..

[139] Wu Q.L., Yang X., Luo J.X., Liu L., Zhou Y., Lu M.H. (2025). Microglia energy metabolism: A new perspective on Alzheimer’s disease treatment. J. Neurol. Sci..

[140] Budny V., Ruminot I., Wybitul M., Treyer V., Barros L.F., Tackenberg C. (2025). Fueling the brain—The role of apolipoprotein E in brain energy metabolism and its implications for Alzheimer’s disease. Transl. Psychiatry.

[141] Meng X., Reis N., Bassik M.C., Pasca S.P. (2026). CRISPR screens in human neural organoids and assembloids. Nat. Protoc..

[142] Guo Y., Zhao X. (2025). CRISPR-based genetic screens in human pluripotent stem cells derived neurons and brain organoids. Cell Tissue Res..

