# Supplementary material for: Stimulus-Based ApoE Alzheimer’s Disease Induction Model Using Microglia-Containing Brain Organoids for Drug Discovery
Source: Cells. 2026 Jul 14;15(14):1266. doi: 10.3390/cells15141266 (PMC13406232; doi:10.3390/cells15141266)
Supplement: Supplementary file 1 [file cells-15-01266-s001.zip › cells-4418234-supplementary.pdf]

Identity	ID #	Cells	Ave transcripts sequenced/cell	Ave genes/cell
APOE3 MG + rApoE4 (24 hrs)	Acute 1	11,607	28,151	6,599
APOE4 MG + rApoE4 (24 hrs)	Acute 2	22,193	29,207	6,726
APOE3 MG + rApoE3 (7 days)	Chronic 1	4,772	19,963	6,050
APOE3 MG + rApoE3 +Aß (7 days)	Chronic 2	10,898	19,355	5,970
APOE4 MG + rApoE4 (7 days)	Chronic 3	10,251	21,204	6,148
APOE4 MG + rApoE4 + Aß (7 days)	Chronic 4	11,570	21,843	6,203

Table S1. ScRNAseq sample sets and conditions. Identity of the sample condition with relevant statistics. Number of cells, average RNA transcripts sequenced per cell, and average number of genes detected per cell were calculated and extracted using Seurat.

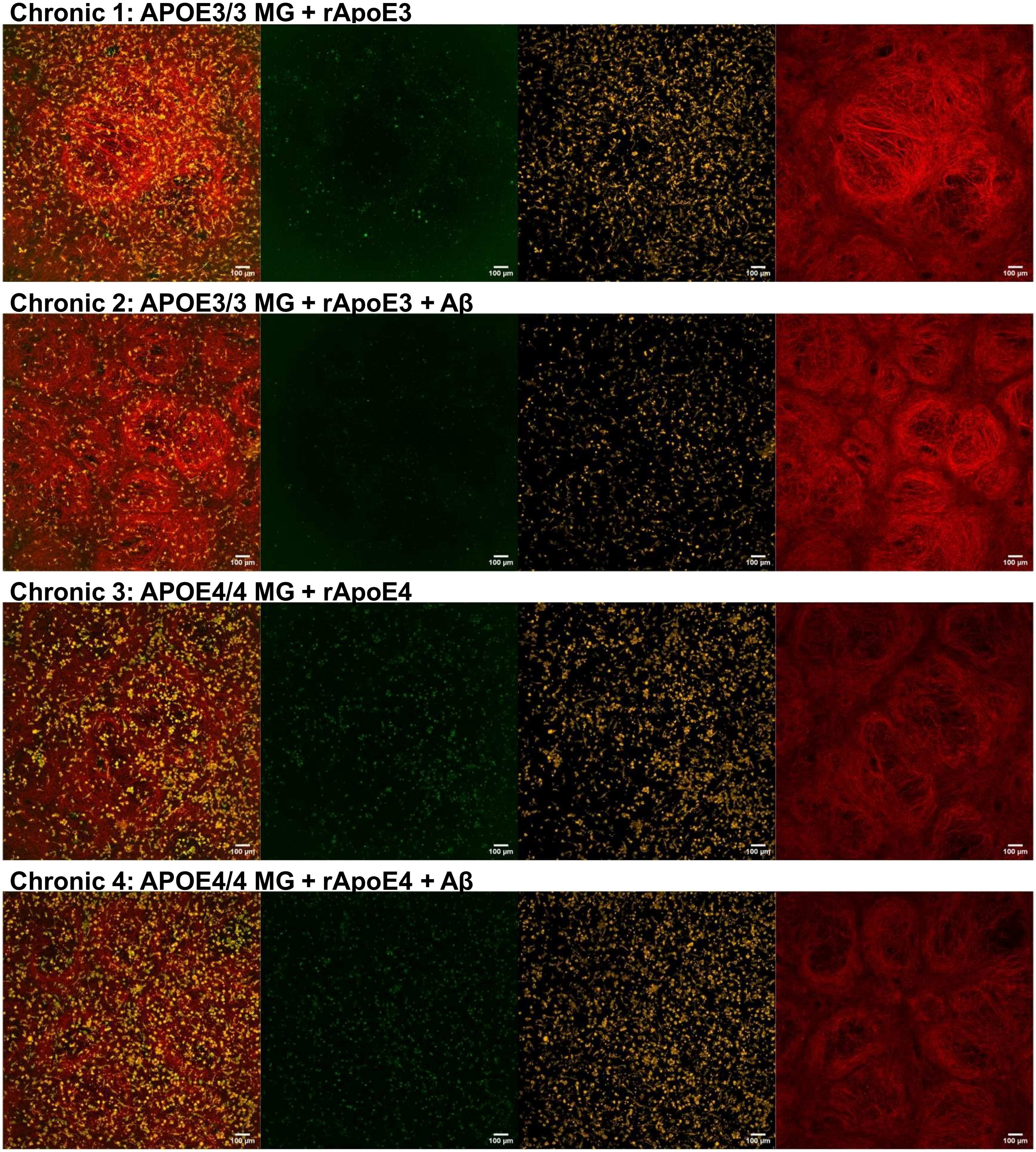

Figure S1. Immunofluorescence staining of APOE3/3 or APOE4/4 MG containing organoids treated with combinations of recombinant ApoE and Aβ after chronic (7 day) induction. Representative maximum intensity projection images comparing control (APOE3/3 MG + rApoE3), control + Aβ (APOE3/3 MG + rApoE3 + Aβ) versus the APOE4/4 MG (APOE4/4 MG + rApoE4), and APOE4/4 MG + Aβ (APOE4/4 MG + rApoE4 + Aβ). Organoids were staining with antibodies specific for IBA1, βIII-Tubulin, and LipidSpot™. Volumetric IF imaging was performed using a 10X confocal. Images shown with channels separated.

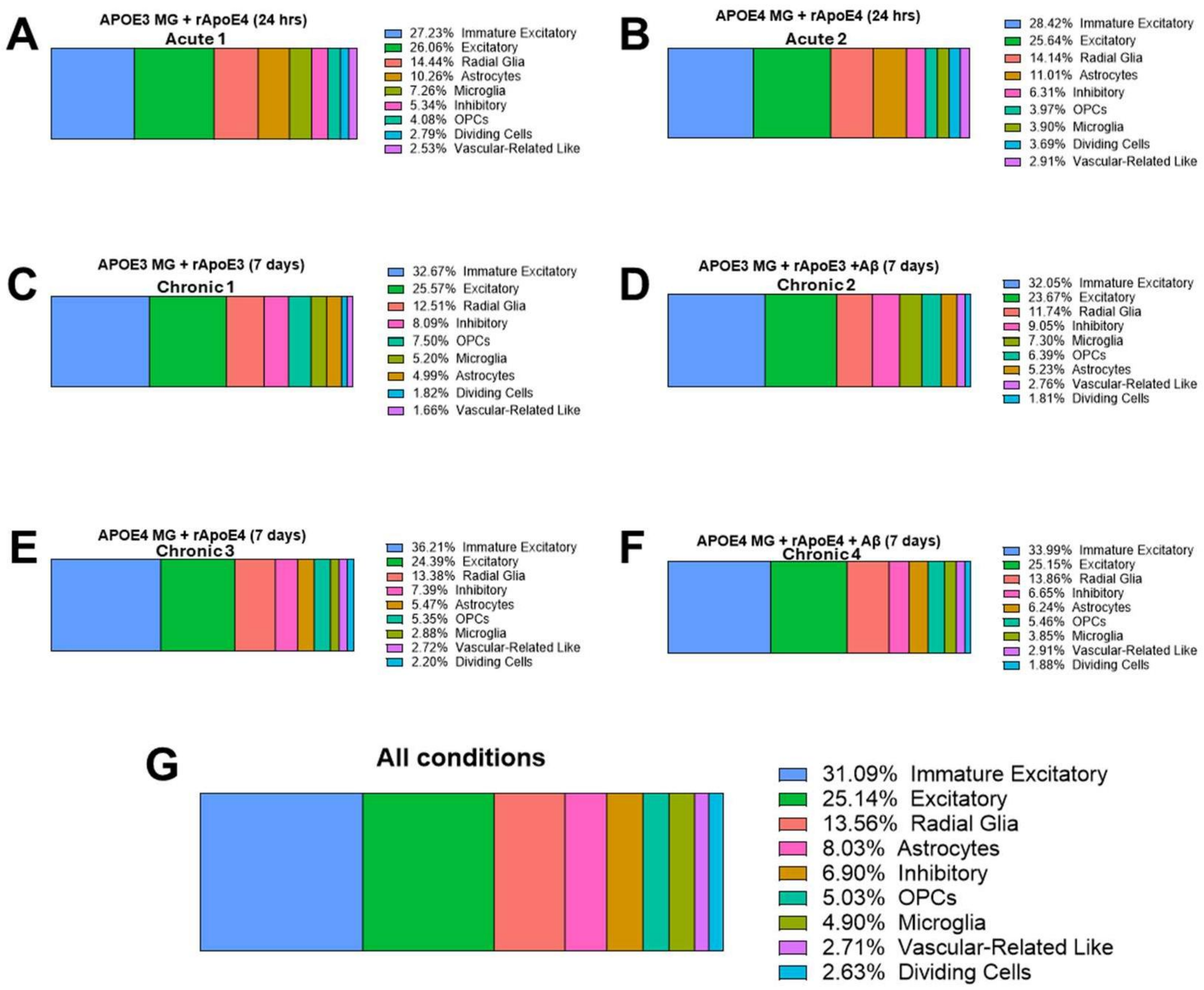

Figure S2. Distribution of cell types detected in the organoid and annotated by scRNAseq. Relative cell populations were quantified from all scRNAseq data sets. Population distributions for the cells detected in organoids containing either (**A**) APOE3/3 or (**B**) APOE4/4 microglia acutely (24 hr) exposed to rApoE4 and organoids containing either (**C-D**) APOE3/3 microglia or (**E-F**) APOE4/4 microglia chronically (7 days) treated to their respective ApoE protein with (**D,F**) or without (**C,E**) seeding of amyloid beta. Refer to Table 1 for full descriptive identities. Cumulative cell type population distribution of all six sets shown in **G**. Values shown are percentages of the total amount of cells analyzed in the scRNAseq experiments. Assigned cell types were annotated as follows: Excitatory (*TLX1, TLX3, SLC17A8, CDH9*), Immature Excitatory (*NEUROG2, NEUROD6*), Inhibitory (*GAD2, SLC6A5, LAMP5, DLX5*), Radial Glia (*AGT, LRP2*), Dividing Cells (*MKI67, DLGAP5*), OPCs (*OLIG1, OLIG2, SOX10*), Vascular-Related Like (*MGP, TEK*), Astrocytes (*AQP4, CP*), and Microglia (*C1QC, CSF1R*).

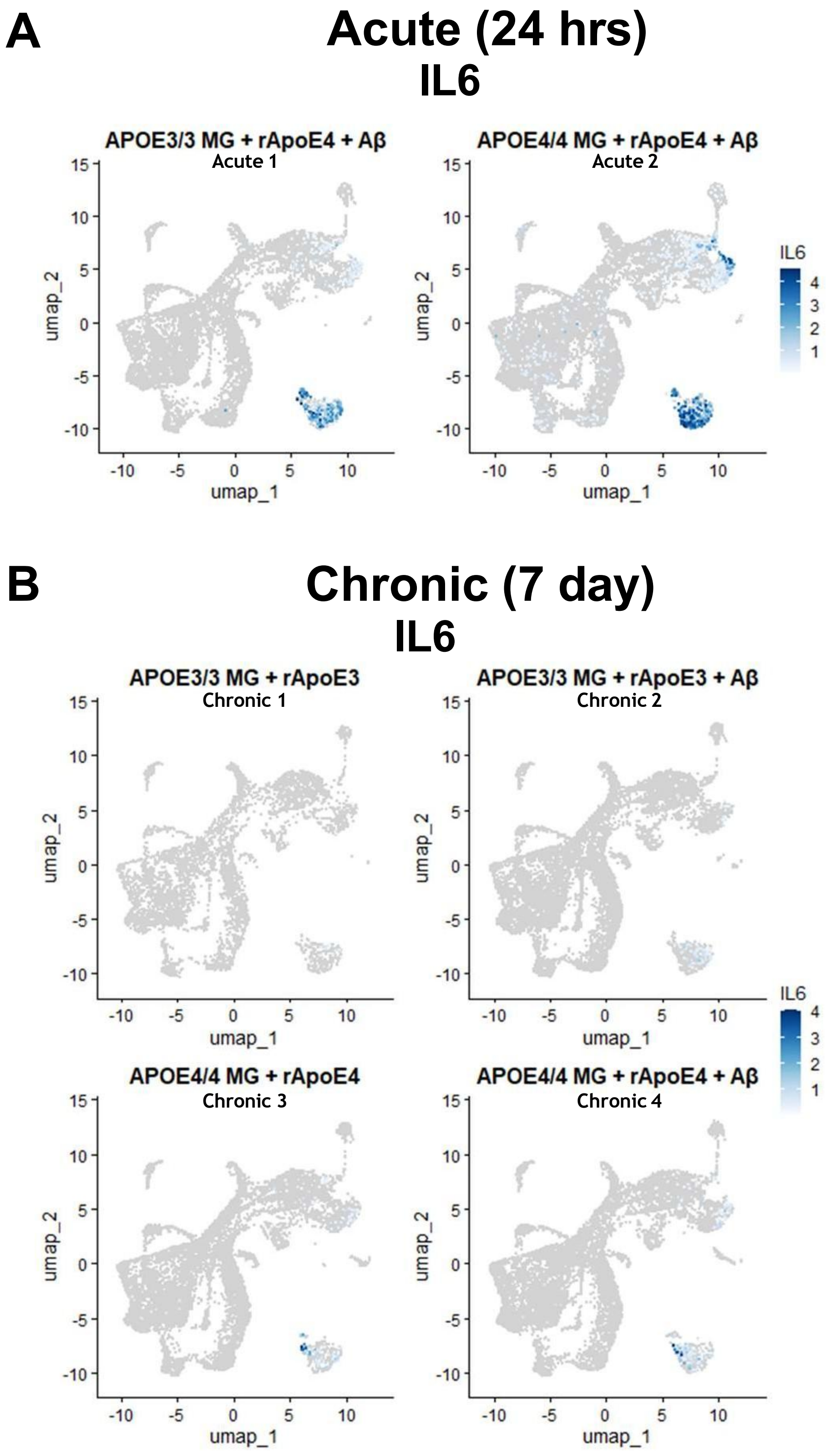

Figure S3. Neuroimmune organoids containing either APOE3/3 or APOE4/4 microglia exhibit acute upregulation of IL6. (**A-B**) UMAP visualization of the scRNAseq data set colored by Log2 expression of IL6. **A**) Proinflammatory cytokine IL6 shows higher gene expression in organoids containing APOE4/4 microglia chronically exposed to rApoE4. **B)** Organoids containing either APOE3/3 or APOE4/4 microglia both show upregulation of IL6 when exposed to rApoE4 after 24 hrs. Clustering and visualization performed using Seurat.

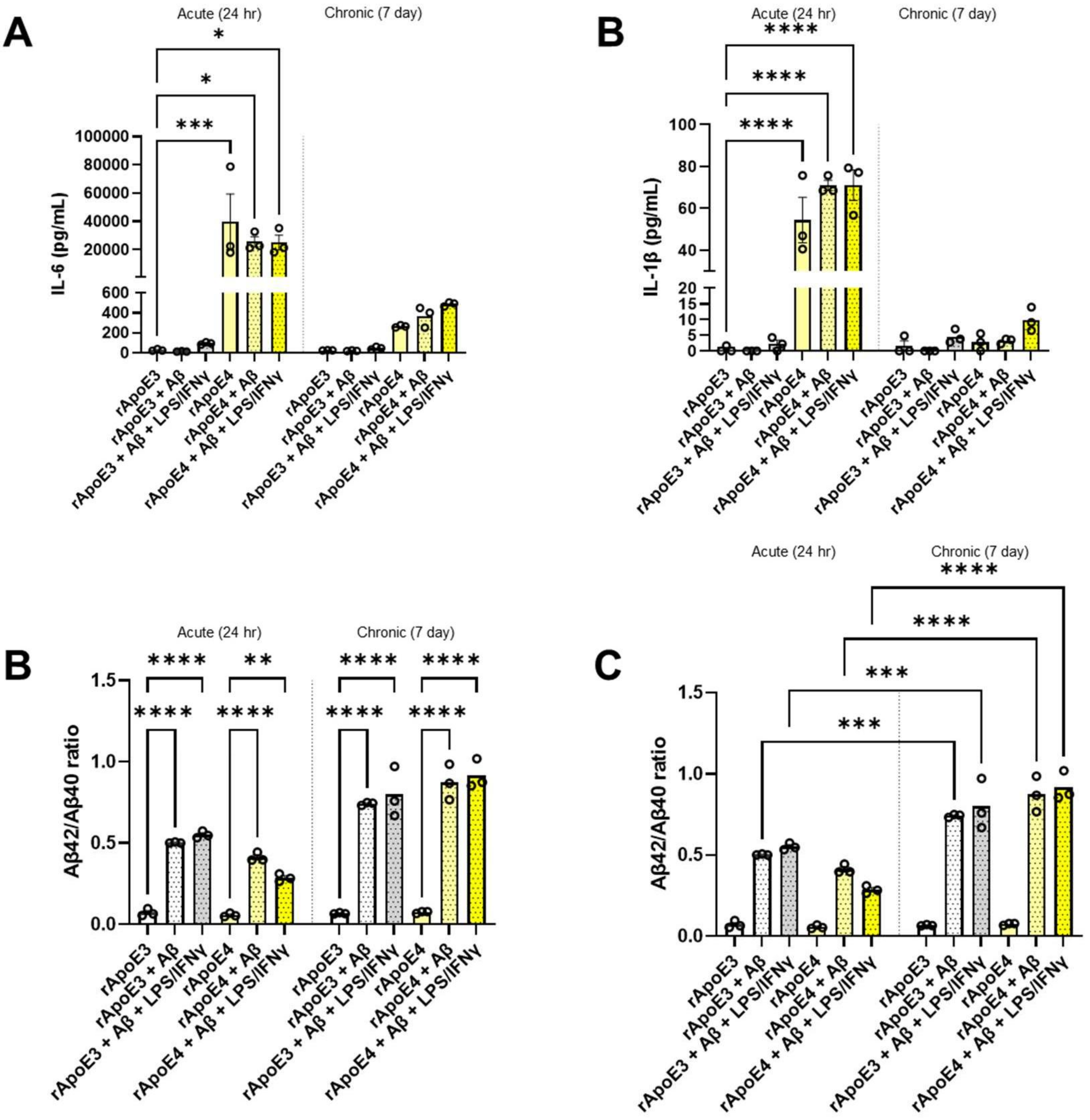

Figure S4. AD relevant IL-6, IL-1ß, and Aβ42/40 ratio over time A) Expanded data from rApoE4 treated organoids (proteomic multiplex experiment shown in Fig 6, B) shows robust acute upregulation of **A**) IL-6 and **B**) IL-1ß. **C)** Expanded data from organoids seeded with Aβ (Fig 7, B) demonstrating that Aβ42/40 ratio is significantly increased relative to untreated controls **(D)** In organoids seeded with Aβ the Aβ42/40 ratio is significantly increased over the course of 7 days. Human Aβ detection performed using Luminex panel (3 organoids per conditions). Statistics performed using one-way ANOVA and Šídák's post hoc test to rApoE3 treated controls, p-value levels of significance are as follows: *, p≤0.05; **, p≤0.01; ***, p≤0.001; ****, p≤0.0001.

	Conditions																	
	Acute (24hr)																	
	rApoE3			rApoE3 + Aβ			rApoE3 + Aβ + LPS/IFNγ			rApoE4			rApoE4 + Aβ			rApoE4+ Aβ + LPS/IFNγ		
Protein (pg/mL)	Mean	SE	p-value	Mean	SE	p-value	Mean	SE	p-value	Mean	SE	p-value	Mean	SE	p-value	Mean	SE	p-value
EGF	6.81	4.98	N/A	9.93	0.45	5.24E-01	11.40	1.33	3.78E-01	17.27	1.25	1.13E-01	15.39	1.79	1.57E-01	24.31	1.54	3.67E-02
Eotaxin	267.95	35.50	N/A	289.17	19.47	6.35E-01	457.75	18.17	1.79E-02	2590.51	354.82	2.17E-02	3971.37	1424.98	1.22E-01	4283.43	472.59	1.32E-02
FGF-2	129.15	13.31	N/A	100.40	23.80	3.66E-01	76.68	5.64	4.29E-02	126.57	6.60	8.73E-01	157.29	30.57	4.66E-01	144.99	14.27	4.63E-01
FLT-3L	4.28	0.30	N/A	3.29	0.13	6.62E-02	12.35	0.32	5.18E-05	13.31	1.45	2.12E-02	13.81	0.13	1.90E-04	19.19	1.29	5.37E-03
Fractalkine	34.47	5.53	N/A	28.89	17.85	7.48E-01	57.27	19.27	3.59E-01	100.91	9.55	7.58E-03	77.45	4.51	4.35E-03	286.29	14.89	1.31E-03
G-CSF	6.49	1.26	N/A	7.89	0.96	4.31E-01	8.37	1.96	4.73E-01	399.75	57.80	2.09E-02	456.74	73.26	2.54E-02	3328.45	980.54	7.72E-02
GM-CSF	0.00		N/A	0.00			0.00			0.00			0.00			0.00		
GROα	31.13	4.82	N/A	23.08	1.69	2.31E-01	249.55	22.90	8.55E-03	4979.48	185.85	1.40E-03	4840.91	292.68	3.67E-03	5257.86	143.17	7.40E-04
IFNα2	13.73	1.09	N/A	14.32	1.66	7.82E-01	22.71	3.02	8.34E-02	28.58	4.74	8.16E-02	37.61	8.78	1.11E-01	56.37	2.03	3.06E-04
IFNγ	10.66	0.62	N/A	10.71	0.35	9.45E-01	4740.01	413.33	7.55E-03	12.78	0.05	7.44E-02	11.55	0.32	2.89E-01	5978.30	705.54	1.37E-02
IL-1α	1.99	0.82	N/A	1.22	0.18	4.48E-01	2.09	0.23	9.16E-01	61.51	10.16	2.73E-02	141.91	29.43	4.14E-02	34.84	5.70	2.68E-02
IL-1β	0.57	0.98	N/A	0.00		4.23E-01	2.19	1.53	3.26E-01	54.41	10.83	3.78E-02	70.92	2.42	6.97E-04	71.09	7.22	9.97E-03
IL-1RA	14.79	6.88	N/A	5.13	0.85	2.95E-01	17.67	1.01	7.17E-01	451.43	51.98	1.27E-02	653.69	39.86	3.11E-03	202.11	30.31	2.08E-02
IL-2	1.01	0.13	N/A	1.20	0.04	2.64E-01	0.97	0.11	8.24E-01	2.82	0.50	6.08E-02	3.08	0.30	1.10E-02	4.46	0.55	1.99E-02
IL-3	2.76	0.57	N/A	2.11	0.25	3.81E-01	3.09	0.17	6.30E-01	2.67	0.17	8.96E-01	3.13	0.19	5.87E-01	4.37	0.24	9.02E-02
IL-4	8.18	0.15	N/A	8.21	0.02	8.48E-01	8.34	0.08	4.19E-01	8.30	0.20	6.56E-01	7.63	0.14	5.53E-02	5.89	0.12	3.94E-04
IL-5	0.25	0.02	N/A	0.21	0.02	2.57E-01	0.26	0.02	8.51E-01	0.30	0.01	1.82E-01	0.27	0.04	7.14E-01	0.52	0.07	4.90E-02
IL-6	28.40	4.97	N/A	15.13	1.50	1.06E-01	95.96	6.96	2.08E-03	39790.98	19564.98	1.79E-01	25607.67	3622.98	1.95E-02	25009.63	5245.16	4.14E-02
IL-7	0.00		N/A	0.00			3.10	0.15	2.25E-03	0.29	0.28	3.38E-01	0.09	0.15	4.23E-01	2.69	0.14	2.88E-03
IL-8	370.40	88.26	N/A	217.39	12.20	2.23E-01	738.67	138.11	9.98E-02	69097.03	20439.68	7.82E-02	48740.72	10308.94	4.25E-02	102782.41	17894.54	2.92E-02
IL-9	6.95	4.57	N/A	3.98	6.89	6.15E-01	11.86	2.46	3.43E-01	14.13	0.56	1.92E-01	15.63	0.88	1.39E-01	18.42	0.57	8.81E-02
IL-10	17.94	1.73	N/A	10.76	2.34	7.42E-02	4.97	0.50	1.20E-02	10495.25	1571.18	2.18E-02	15038.93	753.19	2.50E-03	636.07	136.84	4.57E-02
IL-12p40	17.65	2.25	N/A	17.98	1.28	9.05E-01	78.51	45.41	3.12E-01	9986.91	641.12	4.11E-03	15686.49	788.34	2.52E-03	72519.07	5681.56	6.09E-03
IL-12p70	1.60	0.28	N/A	1.88	0.21	4.73E-01	6.81	4.04	3.25E-01	4.95	0.91	5.66E-02	5.71	0.86	3.12E-02	885.89	261.92	7.76E-02
IL-13	94.85	1.44	N/A	99.19	1.71	1.26E-01	101.99	2.12	5.71E-02	114.00	5.54	6.62E-02	109.22	3.04	2.60E-02	108.15	3.27	3.91E-02
IL-15	3.21	0.37	N/A	2.41	0.37	2.04E-01	32.07	2.71	7.82E-03	15.51	0.87	1.59E-03	18.51	1.13	2.72E-03	45.79	0.07	4.64E-05
IL-17A	0.00		N/A	0.00			0.21	0.37	4.23E-01	0.33	0.26	2.53E-01	1.30	0.24	3.14E-02	3.71	0.26	4.77E-03
IL-17E/IL-25	15.51	3.93	N/A	20.07	4.02	4.63E-01	18.66	5.74	6.77E-01	35.15	6.92	8.56E-02	38.91	2.04	1.32E-02	44.63	5.60	1.65E-02
IL-17F	4.43	0.12	N/A	4.27	0.73	8.55E-01	8.24	1.46	1.19E-01	9.91	0.47	4.87E-03	10.98	2.16	9.29E-02	18.96	0.33	1.17E-04
IL-18	1.87	1.17	N/A	0.78	0.00	4.47E-01	1.12	0.15	5.85E-01	1.58	0.09	8.25E-01	1.57	0.10	8.17E-01	2.53	0.11	6.32E-01
IL-22	191.20	5.65	N/A	175.58	5.06	1.09E-01	179.69	4.34	1.86E-01	196.81	3.58	4.57E-01	192.06	12.22	9.53E-01	162.47	5.92	2.48E-02
IL-27	47.49	4.57	N/A	38.94	2.74	1.99E-01	47.36	1.84	9.80E-01	233.63	13.24	2.33E-03	209.35	15.90	6.08E-03	138.12	0.93	1.80E-03
IP-10	252.70	28.44	N/A	85.93	6.98	2.26E-02	1118.57	34.58	5.54E-05	1351.40	93.58	4.16E-03	1402.23	106.10	5.59E-03	1451.94	49.59	1.59E-04
M-CSF	464.27	27.79	N/A	397.28	17.27	1.24E-01	770.60	8.61	4.73E-03	740.58	29.42	2.44E-03	727.79	22.53	2.13E-03	770.59	3.14	7.53E-03
MCP-1	5901.88	1386.40	N/A	7676.81	99.97	3.29E-01	11072.61	451.82	5.35E-02	12722.89	2721.40	1.12E-01	20879.24	3372.28	3.29E-02	10886.10	2034.92	1.22E-01
MCP-3	16.11	0.49	N/A	12.33	0.53	6.52E-03	67.24	19.94	1.24E-01	3355.35	645.53	3.54E-02	3604.27	290.28	6.48E-03	1527.65	110.68	5.32E-03
MDC	1110.22	485.67	N/A	198.44	41.23	2.01E-01	171.81	53.47	1.92E-01	3864.47	2091.44	3.17E-01	1040.74	77.42	9.00E-01	1231.59	52.75	8.27E-01
MIG/CXCL9	19.22	2.05	N/A	13.12	1.32	7.76E-02	7746.74	455.60	3.46E-03	220.47	16.61	6.12E-03	279.50	19.73	5.30E-03	7483.04	600.36	6.41E-03
MIP-1α	0.00		N/A	0.00			13.03	5.06	1.23E-01	1322.16	2290.05	4.23E-01	3199.18	1970.97	1.85E-01	1286.39	2228.09	4.23E-01
MIP-1β	217.47	23.16	N/A	175.04	6.41	2.03E-01	466.68	61.46	4.22E-02	OOR >			OOR >			OOR >		
PDGF-AA	524.15	52.24	N/A	417.75	31.38	1.71E-01	424.41	31.01	1.92E-01	840.34	12.74	2.12E-02	805.08	45.16	1.59E-02	955.59	57.79	5.35E-03
PDGF-AB/BB	79.91	4.81	N/A	95.60	17.30	4.64E-01	111.16	13.20	1.29E-01	129.59	4.52	1.70E-03	162.57	5.40	3.59E-04	290.81	26.87	1.37E-02
RANTES	3.77	0.45	N/A	3.63	0.85	8.88E-01	10.98	1.15	1.48E-02	1253.11	155.99	1.52E-02	2740.03	247.43	8.08E-03	10593.54	3447.59	9.17E-02
sCD40L	48.80	5.35	N/A	47.99	1.98	8.97E-01	44.65	11.61	7.68E-01	135.97	9.21	2.95E-03	140.81	6.97	6.53E-04	178.60	8.68	6.16E-04
TGFα	2.07	0.75	N/A	3.09	0.80	4.06E-01	5.43	0.21	3.87E-02	10.43	1.04	3.90E-03	11.22	0.49	1.09E-03	15.13	1.67	7.33E-03
TNFα	12.33	3.65	N/A	5.15	0.14	1.87E-01	24.81	2.49	5.50E-02	1826.86	126.09	4.77E-03	2586.73	150.54	3.39E-03	5986.15	467.65	6.07E-03
TNFβ	130.41	5.46	N/A	122.20	5.90	3.65E-01	125.05	4.88	5.06E-01	143.88	3.20	1.17E-01	113.63	6.14	1.12E-01	88.63	2.71	6.83E-03
VEGF-A	4980.95	354.97	N/A	4449.67	304.81	3.21E-01	4025.65	135.51	1.01E-01	4259.66	104.42	1.72E-01	2942.89	205.50	1.34E-02	1352.64	24.53	9.18E-03
6CKine	17.19	0.57	N/A	6.61	2.01	2.68E-02	6.47	5.36	1.32E-01	10.17	7.04	3.47E-01	11.05	6.88	3.88E-01	15.99	1.17	4.25E-01
BCA-1	13.49	1.20	N/A	12.49	2.06	7.01E-01	10.85	0.98	1.65E-01	171.06	17.18	1.14E-02	201.98	11.86	3.65E-03	26.98	0.57	2.45E-03
CTACK	0.65	0.16	N/A	0.52	0.09	5.20E-01	0.31	0.03	1.57E-01	0.63	0.24	9.32E-01	0.76	0.32	7.85E-01	0.87	0.22	4.81E-01
ENA-78	138.81	31.97	N/A	103.73	11.74	3.91E-01	173.78	19.15	4.12E-01	5824.66	335.11	3.22E-03	7416.14	548.86	5.51E-03	5631.77	279.46	2.31E-03
Eotaxin-2	323.55	92.62	N/A	84.85	8.63	1.22E-01	15.06	1.81	7.95E-02	347.53	59.79	8.40E-01	344.58	82.59	8.74E-01	87.30	22.86	1.18E-01
Eotaxin-3	0.00		N/A	0.00			0.00			0.00			0.00			0.00		
I-309	4.88	2.16	N/A	1.20	0.10	2.30E-01	6.99	2.33	5.43E-01	73.87	5.27	2.15E-03	83.81	13.34	2.49E-02	71.00	6.04	4.12E-03
IL-16	20.11	1.86	N/A	15.86	2.75	2.78E-01	9.89	0.99	1.59E-02	13.51	0.86	5.26E-02	12.21	0.59	4.07E-02	7.23	0.73	1.14E-02
IL-20	123.55	90.28	N/A	8.68	15.03	2.59E-01	82.97	59.88	6.74E-01	38.56	66.79	3.82E-01	20.56	35.61	2.95E-01	41.12	25.18	3.81E-01
IL-21	0.00			0.00			0.00			0.00			0.00			0.00		
IL-23	34.26	13.32	N/A	21.13	5.80	4.38E-01	14.55	4.39	2.74E-01	652.10	49.86	4.14E-03	1072.67	104.31	9.07E-03	3243.39	662.79	4.01E-02
IL-28A	1.29	0.13	N/A	0.17	0.11	2.98E-03	0.09	0.15	2.32E-03	0.43	0.38	9.53E-02	0.35	0.60	9.85E-02	0.61	1.05	3.75E-01
IL-33	0.00		N/A	0.00			0.00			0.00			0.00			0.00		
LIF	6.65	1.20	N/A	6.07	0.27	6.81E-01	4.55	0.19	2.22E-01	9.13	0.44	1.65E-01	7.64	1.95	6.91E-01	38.83	9.34	7.27E-02
MCP-2	10.73	0.99	N/A	6.36	0.00	4.79E-02	951.45	197.89	4.15E-02	3003.25	171.03	3.25E-03	2858.43	115.42	1.64E-03	4362.43	596.92	1.83E-02
MCP-4	22.73	4.02	N/A	3.76	0.00	4.21E-02	411.44	59.35	2.21E-02	198.18	15.12	4.83E-03	180.54	22.63	1.75E-02	119.27	4.98	1.49E-04
MIP-1δ	11.31	1.63	N/A	9.27	0.41	3.38E-01	10.91	0.71	8.35E-01	10.50	2.16	7.80E-01	12.95	0.81	4.36E-01	186.97	8.33	1.62E-03
SCF	7.31	0.25	N/A	6.75	0.20	1.58E-01	5.16	0.28	4.76E-03	7.17	0.53	8.26E-01	6.75	0.57	4.38E-01	4.41	0.49	1.42E-02
SDF-1α+β	124.09	16.90	N/A	151.47	17.90	3.29E-01	115.57	6.22	6.74E-01	164.87	15.30	1.49E-01	226.10	22.03	2.39E-02	134.97	7.59	6.01E-01
TARC	0.21	0.02	N/A	0.17	0.02	1.99E-01	0.10	0.00	4.22E-02	0.50	0.06	2.49E-02	0.47	0.13	1.75E-01	0.14	0.02	9.28E-02
TPO	7.03	0.48	N/A	5.92	1.68	5.83E-01	6.20	0.00	2.27E-01	5.91	2.16	6.61E-01	5.92	0.73	2.84E-01	6.47	2.26	8.30E-01
TRAIL	1.42	0.08	N/A	1.02	0.07	2.02E-02	2.18	0.04	3.69E-03	1.70	0.21	3.17E-01	1.60	0.09	2.03E-01	7.28	0.18	1.41E-04
TSLP	0.37	0.03	N/A	0.28	0.04	1.61E-01	0.40	0.04	5.40E-01	0.43	0.05	3.58E-01	0.44	0.02	1.15E-01	0.40	0.04	5.40E-01

Table S2. Expanded data from proteomic multiplex experiment following acute (24 hr) rApoE4 AD induction, shown in heatmap Fig 6, B and graphs in Supplemental Figure 4, A-B. Values and statistics are reported as mean, standard error (pg/mL), and p-value based on unpaired two-tailed t-test to the rApoE3 treated control (n=3 per condition, values below the detectable limit were input as zero, values out of range above the standard curve (logistic regression) are listed as >OOR). Conditions that were statistically significant (p≤0.05) are colored gray within the measurement rows.

	Conditions																	
	Chronic (7 day)																	
	rApoE3			rApoE3 + Aβ			rApoE3 + Aβ + LPS/IFNγ			rApoE4			rApoE4 + Aβ			rApoE4+ Aβ + LPS/IFNγ		
Protein (pg/mL)	Mean	SE	p-value	Mean	SE	p-value	Mean	SE	p-value	Mean	SE	p-value	Mean	SE	p-value	Mean	SE	p-value
EGF	10.45	3.59	N/A	11.83	2.24	7.64E-01	12.57	0.88	6.19E-01	13.23	0.86	5.23E-01	9.95	1.96	9.11E-01	15.56	1.64	2.91E-01
Eotaxin	99.76	25.34	N/A	148.70	53.18	4.70E-01	93.21	25.12	8.63E-01	154.01	15.40	1.56E-01	249.17	40.78	4.56E-02	218.25	95.12	3.39E-01
FGF-2	77.66	15.27	N/A	59.83	7.90	3.76E-01	66.97	12.02	6.13E-01	76.74	13.05	9.66E-01	100.91	13.27	3.16E-01	111.32	9.33	1.48E-01
FLT-3L	3.59	0.27	N/A	3.55	0.43	9.41E-01	11.67	0.91	8.03E-03	10.52	0.29	6.56E-05	11.19	0.32	6.30E-05	12.19	0.21	2.79E-05
Fractalkine	61.07	52.25	N/A	13.33	23.09	3.81E-01	76.31	11.87	7.59E-01	78.16	10.23	7.31E-01	54.20	18.10	8.93E-01	131.39	13.75	2.36E-01
G-CSF	4.96	1.11	N/A	2.71	0.46	1.69E-01	2.53	0.42	1.49E-01	9.62	0.90	3.29E-02	13.76	2.48	5.34E-02	13.97	1.95	2.50E-02
GM-CSF	0.00		N/A	0.00			0.00			0.00			10.73	10.24	3.28E-01	0.00		
GROα	26.32	2.43	N/A	23.55	2.49	4.71E-01	114.74	6.74	2.60E-03	2031.50	158.35	6.17E-03	2567.67	252.37	9.71E-03	2532.70	157.39	3.91E-03
IFNα2	16.27	5.28	N/A	16.35	1.52	9.90E-01	20.85	1.72	4.83E-01	16.29	2.96	9.98E-01	18.35	0.52	7.33E-01	32.09	2.79	7.60E-02
IFNγ	10.67	1.16	N/A	12.15	0.48	3.31E-01	4804.39	326.36	4.60E-03	12.33	0.91	3.27E-01	11.91	1.97	6.23E-01	5570.77	471.87	7.13E-03
IL-1α	1.87	0.83	N/A	0.85	0.12	3.44E-01	1.35	0.25	6.06E-01	2.69	0.68	4.90E-01	2.90	0.25	3.40E-01	4.41	0.72	8.32E-02
IL-1β	1.61	2.78	N/A	0.00		4.23E-01	4.53	1.26	2.30E-01	2.77	1.96	6.34E-01	3.21	0.48	4.26E-01	9.85	2.19	4.33E-02
IL-1RA	4.41	0.35	N/A	4.09	0.44	6.03E-01	8.53	0.58	6.97E-03	14.18	3.16	8.88E-02	17.35	1.71	1.41E-02	93.55	8.68	9.27E-03
IL-2	1.05	0.75	N/A	0.82	0.25	7.98E-01	1.48	0.13	6.25E-01	2.12	0.47	3.04E-01	1.92	0.33	3.72E-01	4.05	0.68	4.20E-02
IL-3	2.14	0.55	N/A	1.43	0.12	3.30E-01	2.24	0.42	8.93E-01	1.57	0.13	4.11E-01	1.35	0.26	2.91E-01	2.21	0.17	9.09E-01
IL-4	8.27	0.45	N/A	9.24	0.36	1.75E-01	8.68	0.16	4.72E-01	8.17	0.34	8.70E-01	7.93	1.14	7.99E-01	8.41	0.52	8.50E-01
IL-5	0.31	0.09	N/A	0.23	0.02	4.60E-01	0.33	0.02	7.91E-01	0.28	0.01	7.90E-01	0.25	0.02	6.09E-01	0.31	0.02	9.47E-01
IL-6	26.03	1.20	N/A	21.78	2.33	2.04E-01	48.23	7.62	9.70E-02	266.09	7.03	6.33E-04	368.65	59.34	2.87E-02	488.39	12.25	6.32E-04
IL-7	0.77	0.29	N/A	0.59	0.23	6.54E-01	3.32	0.38	7.12E-03	1.84	0.10	5.48E-02	2.27	0.26	1.89E-02	3.99	0.22	1.28E-03
IL-8	291.45	12.80	N/A	285.29	23.44	8.32E-01	627.81	43.58	1.13E-02	12691.93	503.41	1.63E-03	17052.20	1911.59	1.28E-02	14313.27	1893.90	1.78E-02
IL-9	4.07	7.06	N/A	3.88	6.72	9.74E-01	5.75	3.90	7.62E-01	6.77	4.40	6.45E-01	6.36	3.90	6.82E-01	15.09	1.88	9.69E-02
IL-10	9.09	0.96	N/A	7.87	0.58	3.47E-01	7.56	0.48	2.50E-01	1601.02	236.54	2.14E-02	2363.27	131.03	3.08E-03	1830.67	136.30	5.55E-03
IL-12p40	18.36	8.51	N/A	13.43	2.45	6.27E-01	11.35	4.20	5.15E-01	58.14	5.72	2.29E-02	53.43	3.61	3.87E-02	84.28	3.76	7.65E-03
IL-12p70	3.98	1.26	N/A	1.92	0.18	2.42E-01	2.13	0.29	2.76E-01	5.87	2.16	5.00E-01	5.75	2.13	5.22E-01	9.91	2.22	9.81E-02
IL-13	106.22	4.94	N/A	109.17	5.42	7.09E-01	102.52	7.01	6.91E-01	99.66	8.62	5.54E-01	99.10	17.34	7.26E-01	106.45	3.93	9.73E-01
IL-15	4.40	1.24	N/A	4.10	0.33	8.35E-01	18.96	0.60	2.18E-03	9.83	0.81	2.80E-02	9.45	0.57	3.83E-02	25.24	0.17	3.07E-03
IL-17A	0.33	0.57	N/A	0.00		4.23E-01	0.15	0.27	6.66E-01	0.00		4.23E-01	0.00		4.23E-01	0.74	0.64	6.06E-01
IL-17E/IL-25	22.61	4.59	N/A	23.97	3.19	8.21E-01	18.86	1.12	5.02E-01	26.31	4.60	6.00E-01	18.43	1.48	4.63E-01	43.70	5.33	4.11E-02
IL-17F	8.25	0.70	N/A	5.11	0.55	2.61E-02	8.33	0.48	9.36E-01	10.34	1.76	3.62E-01	4.91	2.47	3.07E-01	11.51	1.92	2.26E-01
IL-18	1.26	0.15	N/A	0.95	0.16	2.33E-01	1.23	0.17	9.12E-01	1.23	0.06	8.54E-01	1.17	0.27	7.96E-01	2.31	0.08	8.81E-03
IL-22	212.08	13.20	N/A	222.31	6.88	5.41E-01	208.68	3.68	8.24E-01	193.81	25.60	5.71E-01	190.83	42.97	6.76E-01	201.25	11.01	5.64E-01
IL-27	50.75	5.90	N/A	41.12	1.87	2.39E-01	55.04	3.44	5.72E-01	104.71	7.44	5.51E-03	99.33	10.28	2.33E-02	143.35	12.12	7.06E-03
IP-10	690.17	174.72	N/A	863.32	236.44	5.90E-01	1387.03	78.52	4.06E-02	4538.95	510.29	1.06E-02	3957.49	770.52	4.54E-02	1450.16	156.54	3.22E-02
M-CSF	291.93	41.59	N/A	256.56	24.96	5.15E-01	584.95	9.08	1.60E-02	289.27	24.78	9.59E-01	302.05	26.27	8.49E-01	558.40	8.35	2.01E-02
MCP-1	6303.18	1133.50	N/A	11460.15	1331.54	4.33E-02	9762.40	1508.75	1.46E-01	8332.73	529.52	2.08E-01	13269.79	2571.01	9.70E-02	12533.03	3829.37	2.41E-01
MCP-3	14.53	1.63	N/A	12.99	1.37	5.11E-01	57.37	3.85	3.11E-03	1851.50	137.00	5.51E-03	2982.95	261.04	7.64E-03	5328.85	1438.08	6.61E-02
MDC	452.59	177.23	N/A	416.47	35.78	8.59E-01	491.35	125.14	8.68E-01	93.75	25.80	1.78E-01	46.02	13.10	1.48E-01	56.51	7.96	1.55E-01
MIG/CXCL9	14.97	1.34	N/A	13.17	2.05	5.07E-01	11441.03	456.79	1.59E-03	109.10	14.26	2.14E-02	144.62	18.73	1.98E-02	8232.88	133.00	2.61E-04
MIP-1α	0.00		N/A	0.00			0.00			225.19	26.72	1.38E-02	372.24	41.28	1.21E-02	404.24	15.37	1.44E-03
MIP-1β	134.17	4.10	N/A	104.23	20.44	2.79E-01	566.24	32.78	5.14E-03	1432.21	268.65	4.02E-02	3602.85	1051.24	8.09E-02	2727.19	252.57	6.18E-02
PDGF-AA	330.77	20.67	N/A	338.25	52.36	9.04E-01	255.57	26.27	9.14E-02	554.00	24.96	2.64E-03	683.84	126.53	1.04E-01	455.04	33.48	4.41E-02
PDGF-AB/BB	82.05	9.50	N/A	73.15	4.93	4.66E-01	95.65	1.03	2.88E-01	101.45	2.08	1.73E-01	118.71	7.31	4.10E-02	126.35	8.40	2.57E-02
RANTES	3.71	0.31	N/A	2.87	0.72	3.66E-01	23.83	3.93	3.54E-02	27.33	3.16	1.67E-02	41.11	3.95	1.06E-02	147.15	5.45	1.39E-03
sCD40L	70.82	20.42	N/A	41.36	3.20	2.85E-01	53.27	3.79	4.82E-01	77.10	19.79	8.36E-01	78.30	13.36	7.77E-01	100.62	16.24	3.20E-01
TGFα	4.58	0.53	N/A	2.82	0.14	7.25E-02	5.40	0.77	4.37E-01	6.07	0.87	2.32E-01	5.89	0.24	1.19E-01	8.36	0.71	1.52E-02
TNFα	10.20	0.34	N/A	9.14	0.15	7.39E-02	16.56	1.89	7.37E-02	75.43	4.02	3.57E-03	73.79	5.26	6.58E-03	97.52	12.97	2.13E-02
TNFβ	146.26	7.18	N/A	157.71	9.06	3.81E-01	144.39	5.87	8.51E-01	138.20	17.68	7.05E-01	135.76	48.26	8.49E-01	123.11	3.93	6.37E-02
VEGF-A	6455.89	447.49	N/A	8006.83	554.17	9.80E-02	6211.19	813.01	8.09E-01	6228.19	1731.26	9.09E-01	6447.29	3534.95	9.98E-01	4411.19	387.05	2.68E-02
6CKine	8.49	2.39	N/A	9.88	6.05	8.17E-01	6.11	10.58	7.44E-01	7.08	4.34	7.61E-01	5.07	3.77	4.33E-01	23.83	9.01	2.26E-01
BCA-1	3.98	0.17	N/A	3.74	0.77	7.88E-01	3.29	0.31	1.43E-01	1916.98	487.93	5.93E-02	773.28	286.81	1.15E-01	21230.21	19707.84	3.94E-01
CTACK	0.41	0.03	N/A	0.20	0.09	1.50E-01	0.43	0.01	6.21E-01	0.71	0.11	9.27E-02	0.25	0.12	3.22E-01	1.06	0.26	1.24E-01
ENA-78	109.73	18.67	N/A	107.97	9.32	9.38E-01	83.40	8.09	2.94E-01	8205.81	614.23	5.67E-03	9033.97	617.77	4.73E-03	7226.99	545.32	5.77E-03
Eotaxin-2	173.98	8.07	N/A	175.33	5.16	8.96E-01	118.13	16.40	5.73E-02	14.52	3.49	6.44E-04	8.95	1.07	2.05E-03	10.91	1.67	1.72E-03
Eotaxin-3	0.00		N/A	0.00			0.00			0.00			0.00			5.63	9.75	4.23E-01
I-309	1.50	0.04	N/A	0.78	0.12	1.94E-02	3.69	0.61	6.84E-02	12.09	4.94	1.65E-01	11.67	4.65	1.60E-01	13.37	1.76	2.11E-02
IL-16	11.67	2.78	N/A	10.71	1.90	7.93E-01	7.88	2.56	3.73E-01	14.11	2.73	5.65E-01	6.26	4.22	2.92E-01	14.02	4.49	6.83E-01
IL-20	20.56	35.61	N/A	0.00		4.23E-01	0.00		4.23E-01	0.00		4.23E-01	2.81	4.87	4.80E-01	118.36	78.85	2.65E-01
IL-21	0.00			0.00			0.00			0.00			0.00			0.00		
IL-23	23.31	11.59	N/A	18.93	7.59	7.70E-01	18.94	0.00	7.42E-01	29.90	4.38	6.38E-01	23.32	4.38	1.00E+00	53.91	35.80	4.88E-01
IL-28A	0.00		N/A	0.00			0.09	0.15	4.23E-01	1.21	1.32	3.80E-01	0.00			2.49	2.44	3.38E-01
IL-33	0.00		N/A	0.00			0.00			0.00			0.00			0.29	0.51	4.23E-01
LIF	5.00	0.30	N/A	5.75	0.15	1.12E-01	5.60	0.27	2.10E-01	5.91	0.41	1.54E-01	8.17	2.57	3.42E-01	7.05	1.48	2.98E-01
MCP-2	17.09	2.43	N/A	17.20	3.88	9.82E-01	1914.27	159.09	6.95E-03	2624.11	338.82	1.65E-02	2164.51	254.09	1.37E-02	10216.13	2935.32	7.38E-02
MCP-4	214.37	66.89	N/A	300.72	136.20	6.10E-01	829.14	113.86	1.58E-02	2785.20	523.54	3.72E-02	4063.20	590.27	2.16E-02	3627.11	581.93	2.66E-02
MIP-1δ	8.88	3.33	N/A	10.50	2.16	7.07E-01	7.24	0.00	6.71E-01	9.28	2.04	9.24E-01	8.87	2.94	9.99E-01	19.09	6.44	2.54E-01
SCF	14.66	0.57	N/A	14.05	1.45	7.27E-01	12.04	0.68	4.37E-02	15.02	0.08	5.94E-01	11.30	0.28	1.43E-02	10.47	0.42	5.36E-03
SDF-1α+β	67.63	9.95	N/A	95.49	10.19	1.22E-01	86.28	9.80	2.53E-01	324.09	1.99	1.04E-03	257.87	28.31	1.36E-02	349.78	11.12	5.04E-05
TARC	0.12	0.01	N/A	0.18	0.09	5.75E-01	0.11	0.01	6.49E-01	0.12	0.02	1.00E+00	0.10	0.00	2.25E-01	0.20	0.07	3.73E-01
TPO	2.60	0.28	N/A	4.54	1.66	3.63E-01	4.81	1.54	2.86E-01	6.75	1.46	9.96E-02	3.43	0.55	2.73E-01	11.72	4.71	1.92E-01
TRAIL	0.94	0.04	N/A	1.00	0.09	5.79E-01	1.64	0.07	2.94E-03	1.02	0.07	3.87E-01	0.98	0.08	6.86E-01	1.42	0.41	3.58E-01
TSLP	0.34	0.02	N/A	0.40	0.04	2.88E-01	0.32	0.02	5.19E-01	0.38	0.04	4.38E-01	0.31	0.01	2.32E-01	0.56	0.14	2.65E-01

Table S3. Expanded data from proteomic multiplex experiment following chronic (7 day) rApoE4 AD induction, shown in heatmap Fig 6, B and graphs in Supplemental Figure 4, A-B**.** Values and statistics are reported as mean, standard error (pg/mL), and p-value based on unpaired two-tailed t-test to the rApoE3 treated control (n=3 per condition, values below the detectable limit were input as 0 pg/mL). Conditions that were statistically significant ($p \leq 0.05$) are colored gray within the measurement rows.
